# RSV hijacks cellular protein phosphatase 1 to regulate M2-1 phosphorylation and viral transcription

**DOI:** 10.1371/journal.ppat.1006920

**Published:** 2018-02-28

**Authors:** Charles-Adrien Richard, Vincent Rincheval, Safa Lassoued, Jenna Fix, Christophe Cardone, Camille Esneau, Sergei Nekhai, Marie Galloux, Marie-Anne Rameix-Welti, Christina Sizun, Jean-François Eléouët

**Affiliations:** 1 Unité de Virologie et Immunologie Moléculaires (UR892), INRA, Université Paris-Saclay, Jouy-en-Josas, France; 2 UMR1173, INSERM, Université de Versailles St. Quentin, Montigny le Bretonneux, France; 3 Institut de Chimie des Substances Naturelles, CNRS, Université Paris-Saclay, Avenue de la Terrasse, Gif-sur-Yvette, France; 4 Center for Sickle Cell Disease and Department of Medicine, Howard University, Washington, D. C., United States of America; 5 AP-HP, Laboratoire de Microbiologie, Hôpital Ambroise Paré, Boulogne-Billancourt, France; Thomas Jefferson University, UNITED STATES

## Abstract

Respiratory syncytial virus (RSV) RNA synthesis occurs in cytoplasmic inclusion bodies (IBs) in which all the components of the viral RNA polymerase are concentrated. In this work, we show that RSV P protein recruits the essential RSV transcription factor M2-1 to IBs independently of the phosphorylation state of M2-1. We also show that M2-1 dephosphorylation is achieved by a complex formed between P and the cellular phosphatase PP1. We identified the PP1 binding site of P, which is an RVxF-like motif located nearby and upstream of the M2-1 binding region. NMR confirmed both P-M2-1 and P-PP1 interaction regions in P. When the P–PP1 interaction was disrupted, M2-1 remained phosphorylated and viral transcription was impaired, showing that M2-1 dephosphorylation is required, in a cyclic manner, for efficient viral transcription. IBs contain substructures called inclusion bodies associated granules (IBAGs), where M2-1 and neo-synthesized viral mRNAs concentrate. Disruption of the P–PP1 interaction was correlated with M2-1 exclusion from IBAGs, indicating that only dephosphorylated M2-1 is competent for viral mRNA binding and hence for a previously proposed post-transcriptional function.

## Author summary

Respiratory syncytial virus (RSV) is the leading cause of lower respiratory tract illness in infants. Since no vaccine and no potent antivirals are available against RSV, it is essential to better understand the mechanisms of viral replication to develop new antiviral strategies. Here we have investigated the mechanisms by which two essential components of the viral RNA polymerase machinery, the phosphoprotein P and the M2-1 transcription factor, interact and function. We identified the amino acid residues of P critical for this interaction and showed that they are required for P recruiting M2-1 to cytoplasmic inclusions, where viral polymerase complex proteins concentrate and viral RNA synthesis occurs. We also showed that M2-1 dephosphorylation, required for viral transcription, is achieved by a complex formed between P and the cellular phosphatase PP1. The region of P binding to PP1 is located nearby and upstream of the M2-1 binding domain. This is the first report showing that the phosphoprotein of a negative strand RNA virus can hijack a cellular phosphatase to modulate the phosphorylation state of its partners. These two P regions interacting with M2-1 and PP1 are also potential targets for future antiviral therapy.

## Introduction

Human respiratory syncytial virus (RSV) is the leading cause of severe respiratory tract infections in infants worldwide and the primary cause of infant hospitalization for respiratory infections [1]. In addition, RSV is increasingly recognized as a significant cause of disease in the elderly population and can often be fatal for patients with a compromised immune system [2]. The virus belongs to the *Orthopneumovirus* genus of the *Pneumoviridae* family, order *Mononegavirales* [3]. The RSV genome is a single strand, negative sense RNA of about 15 kb that is packaged by the nucleoprotein (N) and maintained as a left-handed helical N-RNA ribonucleoprotein complex (RNP) [4–6]. This RNP is the template for two distinct activities: RNA replication that generates genomic and antigenomic RNA, which is encapsidated by N immediately after synthesis [7, 8], and RNA transcription that generates 10 capped and poly-adenylated mRNAs, which are not encapsidated by N. Both activities are carried out by the viral RNA-dependent RNA polymerase complex (RdRp) [9]. The viral N, P (phosphoprotein) and L (large polymerase) proteins are the essential components of the RdRp. RSV P is the main cofactor of the large polymerase L protein. In particular, by interacting with L and the RNP, P is essential to properly position the L protein for RNA synthesis [10].

RSV transcription is dependent on a fourth viral protein, M2-1 [11]. The transcriptase complex first engages promoter sequences that lie at the 3’ end of the genome [12]. Transcription proceeds through sequential stop-and-restart events, in which the RdRp recognizes gene start (GS) and gene end (GE) sequences, that flank each gene and direct initiation and termination of transcription, respectively [13]. The transcriptase complex has the propensity to dissociate from the RNP template, but cannot reinitiate at a downstream gene after a premature termination. This leads to a decreasing gradient of mRNA from the 3’ to the 5’ end of the genome [14]. In contrast, the highly processive replicase bypasses GS and GE signals to produce complete genomic and antigenomic RNAs [15]. The exact mechanism of how RdRp differentiates between transcription and replication still remains unknown.

By increasing the processivity of the RdRp complex, RSV transcription antiterminator protein M2-1 prevents premature transcription termination [16, 17]. For this activity, M2-1 has to be recruited to cytoplasmic inclusion bodies (IBs), which contain other components of the RdRp complex, notably N, P and L, and [18–20], [21],[22]. Moreover we recently showed that IBs are a place of viral RNA synthesis and that M2-1 and viral mRNAs concentrate in IB substructures called IB associated granules (IBAGs), from which N, P, L and genomic RNA are absent [20]. M2-1 is a 22 kDa basic protein that forms stable tetramers in solution [23, 24]. Each protomer features an N-terminal zinc finger domain, an α-helical tetramerization motif, and a C-terminal α-helical core domain [24]. M2-1 is an RNA binding protein [25] that binds preferentially to RSV mRNAs and A-rich sequences [26]. RSV M2-1 can also interact with RSV P. We showed previously that substitution of M2-1 residues involved in the M2-1–P interaction prevented the recruitment of M2-1 to IBs, suggesting that formation of a P–M2-1 complex is critical for M2-1 recruitment to IBs [19]. Since M2-1 interacts with RNA and P in a competitive manner through the core domain [19, 23], it is expected that these interactions are regulated in a cyclic manner.

In RSV-infected cells M2-1 exists in different phosphorylation states, resulting in its migration as two major bands in SDS-PAGE [27–29]. The slower-migrating species contains phosphorylated M2-1, whereas the faster-migrating species lacks significant phosphorylation [27]. In RSV infected cells or when co-expressed with P, M2-1 protein remains mainly unphosphorylated, whereas M2-1 is mainly phosphorylated when expressed alone [25]. Using recombinant (unphosphorylated) M2-1 produced in *E*. *coli*, it has also been shown that M2-1 can be phosphorylated *in vitro* by casein kinase I on serines S58 and S61 [26]. Abolishing phosphorylation of these residues by alanine substitution impaired the antitermination function of M2-1 [26]. However, the P–M2-1 interaction appears to be independent of the phosphorylation status of M2-1 [23]. On the other hand, phosphorylated M2-1 has reduced RNA binding capacities [25]. All these data point to the critical role of dynamic and reversible M2-1 phosphorylation for its function in transcription.

Neither P nor M2-1 produced in *E*. *coli* are phosphorylated [23, 30, 31]. These two unphosphorylated proteins interact together and have been used previously to study the P–M2-1 interactions *in vitro* [23, 32]. In P, the region encompassing residues 100–120 and more specifically residues L101, Y102, and F109 were reported to be critical for the M2-1–P interaction and for efficient transcription [33]. It was also shown that P residue T105 is probably involved in M2-1 binding [34]. P can be phosphorylated on several serine and threonine residues with different turnover rates [34–39]. In particular phosphorylation of T108, which occurs with a high-turnover, would prevent M2-1 binding. However, the role of P phosphorylation remains unclear, since phosphorylation is not required for viral transcription or replication [38, 40, 41]. Here we further investigated structural and functional aspects of the M2-1–P interaction. We show that a region encompassing residues 93–110 of P is required for the presence of M2-1 in IBs. We further identified another element, located upstream of this region which is responsible for M2-1 dephosphorylation. We show that this region is involved in the binding of the cellular protein phosphatase-1 (PP1) to P, but not in M2-1 binding, and that the complex formed by P and PP1 is responsible for M2-1 dephosphorylation, a key process for efficient viral transcription.

## Results

### Mapping of M2-1_core_ interaction regions on P by NMR

We previously mapped the interaction surface of RSV P on the core domain of M2-1 (M2-1_core_, residues 58–177) by NMR [19], by observing spectral perturbations, at a residue-specific level, induced by unlabeled P on ^15^N-labeled M2-1_core_. RSV P forms highly stable tetramers [31, 42, 43], with large N- and C-terminal intrinsically disordered regions (IDRs) flanking the oligomerization domain (residues 126–163) [31, 32]. We recently analyzed the propensities of these IDRs to form transient secondary structures and to transiently associate, either with each another or with the N protein, also by NMR [44]. Here we proceeded to determine the M2-1 binding region in P.

**Fig 1:**
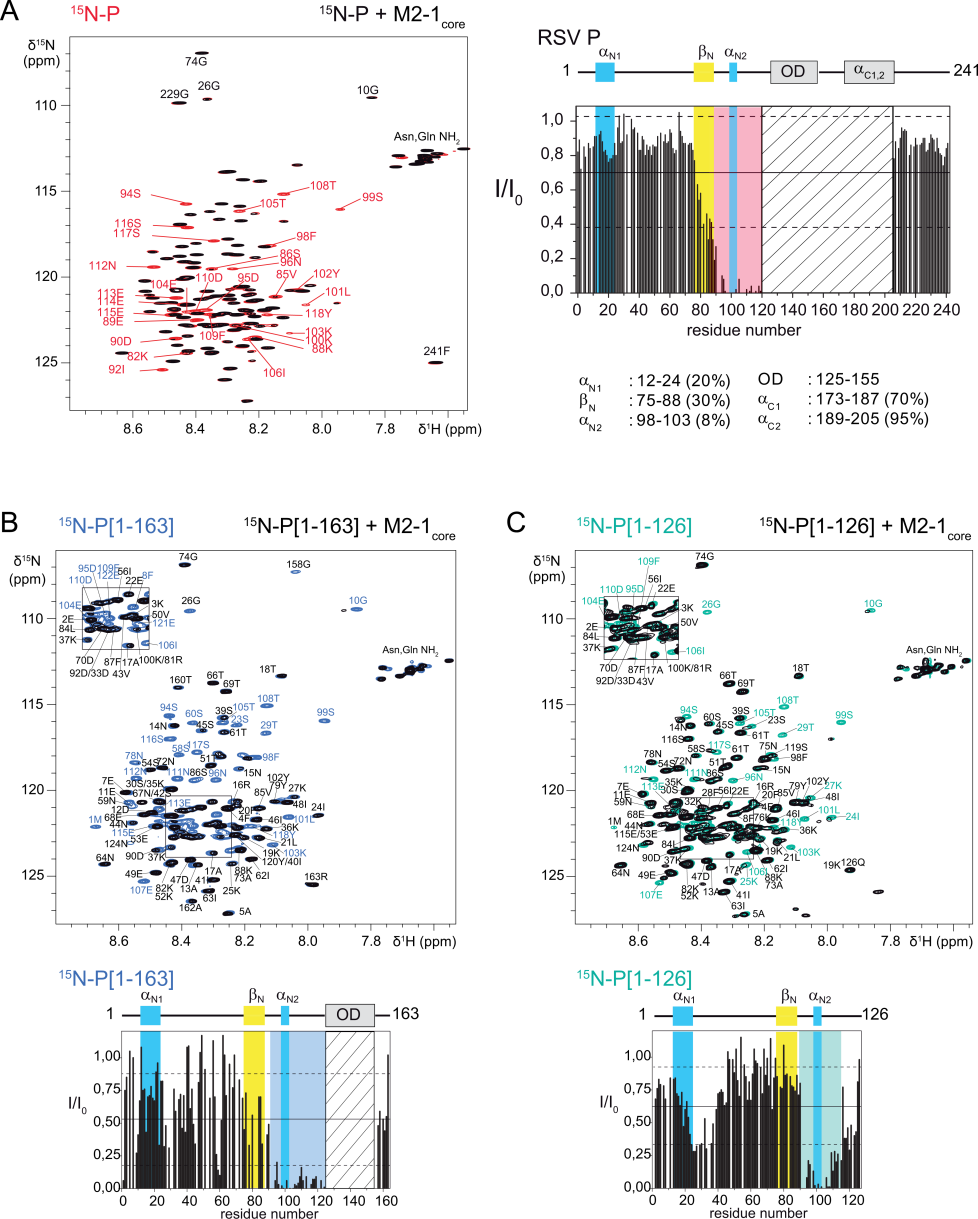
Mapping of the M2-1_core_ interaction regions on P by NMR. (A) Superimposed ^1^H-^15^N HSQC spectra of 35 μM ^15^N-labeled P, alone (red contours) and in the presence of 1 molar equivalent of M2-1_core_ (black contours). Residue-specific assignments are represented by red labels for peaks with an intensity decrease by more than 60% on addition of M2-1_core_. The bar diagram represents the intensity ratios (I/I_0_) measured for each peak. Straight and broken lines represent the mean and mean ± rmsd values over all signals. The position of the oligomerization domain and regions with transient α-helical or β-sheet secondary structure along the sequence of P, previously determined by NMR [44], is indicated in the cartoon on top. The boundaries and maximal propensity of these secondary structures [44] are indicated below. The red background indicates the region around the transient helix α_N2_ where M2-1_core_ induces >60% intensity decrease. The hatched area corresponds to a P region encompassing the OD and C-terminal helices for which peaks cannot be observed due to severe line broadening without M2-1_core_. (B) and (C) Superimposed ^1^H-^15^N HSQC spectra of 35 μM ^15^N-labeled P[1–126] and 75 μM P[1–163], alone (cyan and blue contours) and in the presence of 1 molar equivalent of M2-1_core_ (black contours). Residue-specific assignments are given for all peaks. Insets on the top left give assignments for the crowded central region of the spectra. These spectra were acquired with less resolution in the ^5^N dimension than the spectrum shown in A. Bar diagrams below represent the intensity ratios. The regions around α_N2_ with I/I_0_ < (mean-rmsd) are highlighted with light cyan and blue backgrounds for P[1–126] and P[1–163], respectively.

Both P and M2-1, produced in *E*. *coli*, were unphosphorylated. We compared ^1^H-^15^N spectra of ^15^N-labeled P in the absence and presence of unlabeled M2-1_core_. The presence of M2-1_core_ induced a significant decrease of NMR signal intensities, or line broadening, in a region spanning residues 90–120 in the N-terminal IDR of P, upstream of the oligomerization domain (Fig 1A). This finding is in agreement with the previous localization of residues critical for M2-1 binding [33, 34]. The binding region includes an extremely transient α-helix detected in free P (α_N2_ in Fig 1A) [44]. It is expected that in the M2-1–P complex, the α_N2_ region adopts the molecular tumbling properties of the globular M2-1_core_, which results in increased transverse relaxation and hence line broadening. But this cannot explain that the signal is fully broadened out. A Kd of 3 μM was previously determined for the M2-1–P complex by isothermal titration calorimetry (ITC) [19]. It is compatible with exchange on a μs-ms timescale between free and M2-1_core_-bound P, which contributes to line broadening. Additional broadening probably arises from conformational exchange taking place between partially folded states of the α_N2_ helix, which may all contribute to M2-1_core_ binding.

We also carried out NMR interaction experiments between M2-1_core_ and two ^15^N-labeled P fragments, P[1–126] and P[1–163], which were designed for previous characterization and interaction experiments with N by NMR [44]. They both comprise the 90–120 region. P[1–126] is a monomeric fragment that represents the N-terminal IDR of P. P[1–163] additionally contains the oligomerization domain of P and displays a similar behavior to full-length P, with nearly complete line broadening in the α_N2_ region (Fig 1B). Surprisingly, in P[1–126] a second region is perturbed at the N-terminus of P (residues 23–37) at a lesser extent. This region overlaps with the N^0^-binding site of P, which contains another transient α-helix (α_N1_) [44, 45]. Notably, in N^0^-P interaction experiments using RNA-free N, we had observed the symmetrical scenario: N not only induced line broadening in the N^0^-binding region, but also to a lesser extent in the M2-1-binding region [44]. We cannot rule out that a direct interaction takes place in both cases at a second binding site. However, since we evidenced transient contacts between these two regions in free P [44], the perturbations observed at the second site may indirectly arise from breaking of internal contacts to expose the primary binding site [44]. Finally, from the interaction experiment with P[1–126], it appears that the primary interaction region is probably restricted to residues 90–112, since residues 113–120 are no longer completely broadened out (Fig 1C). The presence of the oligomerization domain in full-length P and P[1–163] can affect nuclear relaxation in this region by restricting motions due to steric hindrance or by promoting an extension of either α_N2_ or the coiled-coil helices of the oligomerization domain.

### P fragment P[93-110] is sufficient for binding M2-1 *in vitro*

**Fig 2:**
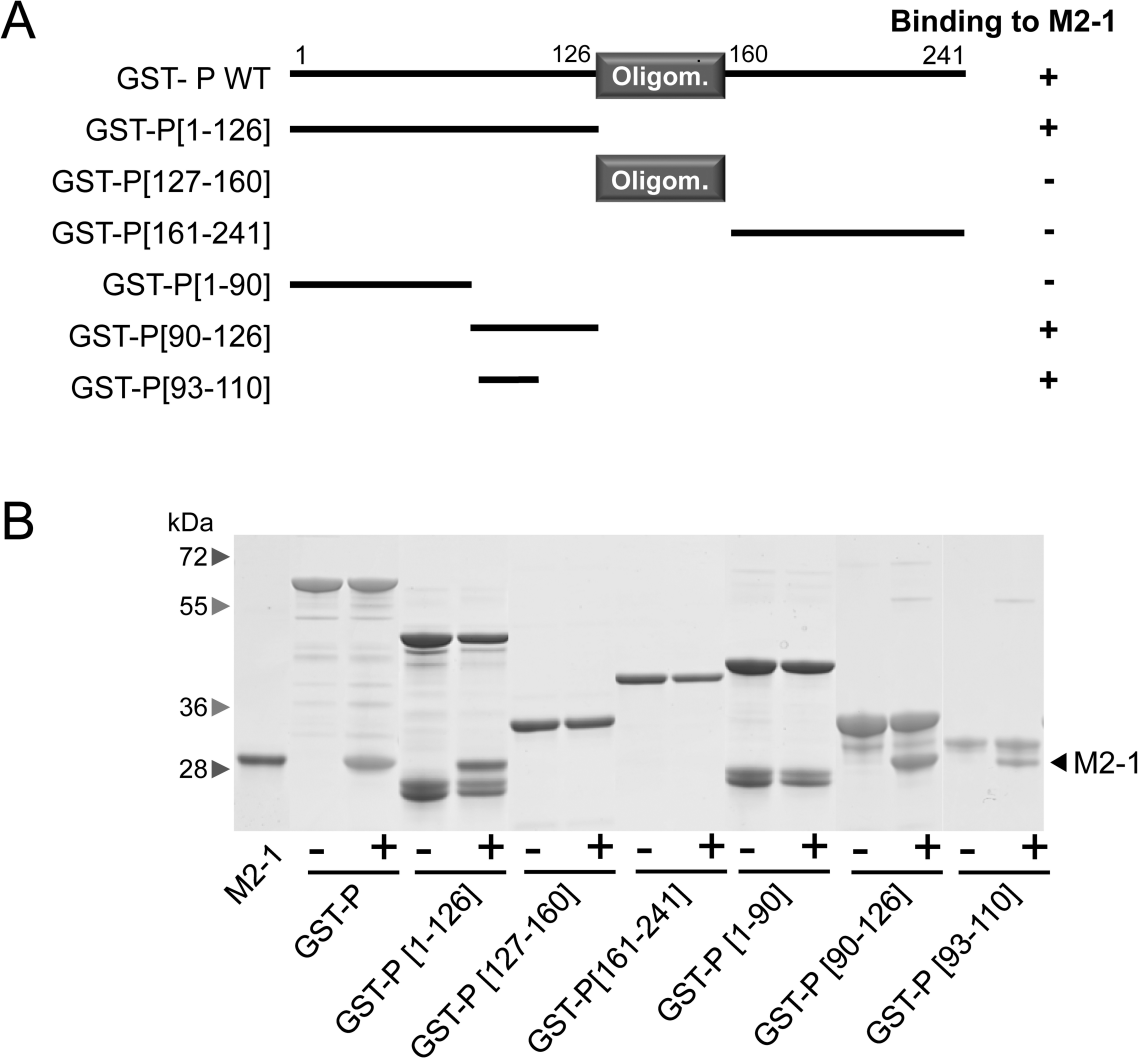
Identification of the M2-1 binding domain on P by GST pull-down. (A) Schematic illustration of the full-length and truncated forms of GST-P used for M2-1 pulldowns in this study. The oligomerization domain of P is represented as a grey box, and numbers indicate amino acid positions. (B) GST-P proteins were purified on glutathione-Sepharose beads and incubated in the presence of M2-1. After extensive washing, the binding of M2-1 was determined by SDS-PAGE and Coomassie blue staining. For each deletion mutant, the ability to interact with M2-1 is summarized on the right in **A**.

To validate our NMR results, we performed GST pulldown assays. Full-length and truncated forms of RSV P were produced as GST-fusion proteins (Fig 2A) and incubated with recombinant His-tagged M2-1 protein. All proteins were expressed in *E*. *coli*. After extensive wash, the complexes were analyzed by SDS-PAGE and Coomassie blue staining. As shown in Fig 2B, M2-1 and P constructs were purified to > 90% homogeneity, except for GST-P[1-126] and GST-P[1-90], for which faster migrating bands were observed in a reproducible manner. Mass spectrometry analysis revealed that these bands correspond to degradation of these P fragments. The results clearly show that M2-1 binding was retained for GST-P, GST-P[1-126], GST-P[90-126] and GST-P[93-110]. However, no M2-1 binding was seen for GST-P[161-241], GST-P[127-160] or GST-P[1-90]. These results, which are in agreement with previous data by Mason et al. [33], demonstrate that P region P93-D110 is sufficient for binding M2-1 *in vitro*.

### Effect of targeted P gene substitutions on M2-1 controlled transcription

**Fig 3:**
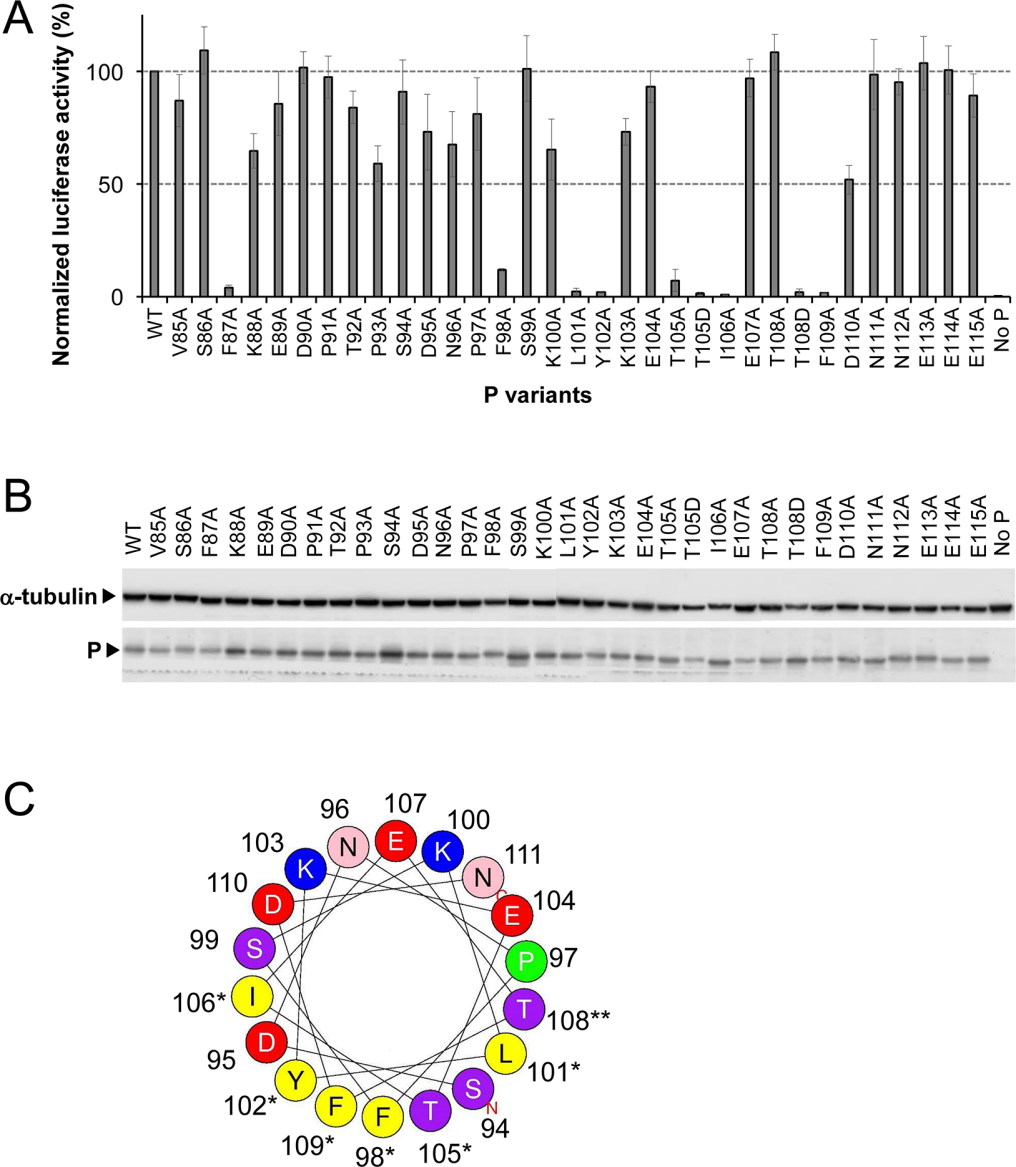
Identification of residues of P critical for RSV polymerase activity. (A) Polymerase activity assay in the presence of P mutants. BSRT7/5 cells were transfected with the RSV minigenome composed of plasmids encoding the WT N, M2-1, and L proteins, the pMT/Luc minigenome, and WT or mutated P proteins, together with pCMV-βGal for transfection standardization. Viral RNA synthesis was quantified by measuring the Luc activity after cell lysis 24 h post-transfection. Each luciferase minigenome activity value was normalized based on β-galactosidase expression and is the average of results from three independent experiments performed in triplicate. Error bars represent standard deviations. (B) Western blot analysis showing efficient expression of P variant proteins in BSRT7/5 cells. (C) Helical wheel representation of the putative α-helix located between residues S94 and N111 of P (HeliQuest online program). Residues critical for M2-1-binding are indicated by a star and the phosphorylatable residue T108 is indicated by two stars. Positively charged residues are in blue, negatively charged residues in red, putative phosphorylated residues in purple and hydrophobic residues in yellow.

In order to identify the P residues critical for P–M2-1 interaction, we used a functional assay based on a firefly luciferase (Luc) reporter RSV minigenome. In this system transcription of the second gene coding for Luc is absolutely dependent on the P–M2-1 interaction [19, 23]. Site-directed mutagenesis was performed on residues V85 to E115, encompassing the P93-D110 region. Ala substitution of seven mainly hydrophobic residues, F87, F98, L101, Y102, T105, I106 and F109, had a drastic effect on RSV transcription (Fig 3A). Western blot shows that the drop in transcription efficiency was not due to a defect in expression of the P variants (Fig 3B). Among these, L101, Y102, T105 and F109 had been shown previously to be involved in the P–M2-1 interaction [33, 34]. The phosphomimetic T108D variant impaired transcription, in contrast to T108A. This is in line with the results of Asenjo et al. who had suggested that phosphorylation of T108 could negatively regulate the P–M2-1 interaction [34]. Our results are thus in agreement with previous data, but highlight the critical role of newly identified residues F87, F98 and I106.

Mutations of P impairing transcription are located in the P93-D110 region, except for F87. The (*i*, *i+3; i*, *i+4*) periodicity of the critical residues F98-F109 of P identified here suggests that M2-1 binding stabilizes the transient α-helix formed in free P [44]. Helical representation of this domain reveals that critical residues F98, L101, Y102, T105, I106 and F109 are located on the same side of the putative helix and form a contiguous surface (Fig 3C).

### Impact of mutations of P on the intracellular localization of M2-1

**Fig 4:**
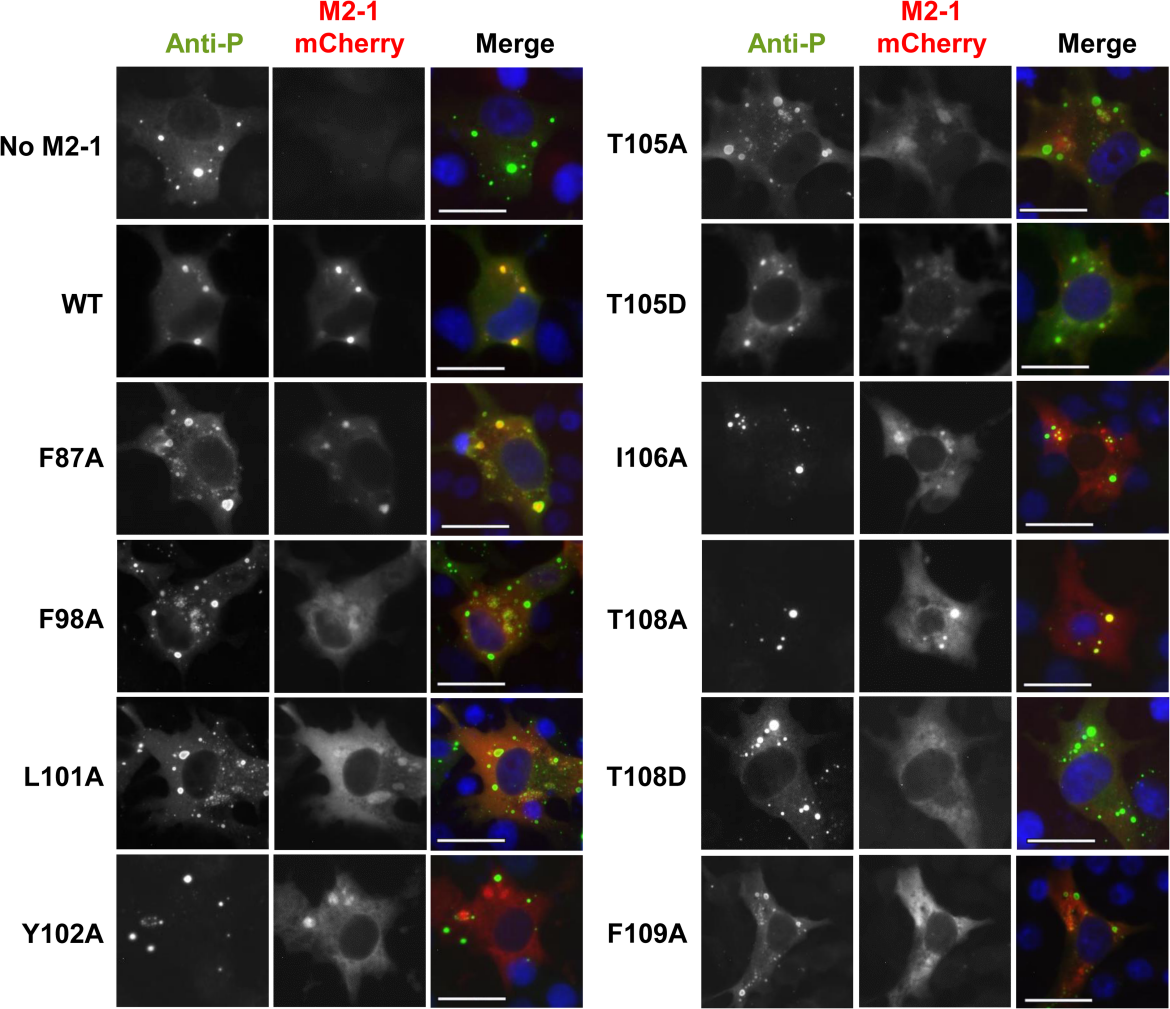
Effects of point mutations targeting the M2-1-binding domain of P on the recruitment of M2-1 to cytoplasmic IBs. BSRT7/5 cells were transfected with pP (WT and variants), pN, and pM2-1–mCherry plasmids. Cells were fixed 24 h after transfection, labeled with anti-P antibody (green), and colocalization of P and M2-1–mCherry (red) was analyzed by fluorescence microscopy. Nuclei were stained with DAPI. Scale bars, 20 μm.

When co-expressed in the absence of other viral proteins, RSV P and N proteins induce the formation of cytoplasmic IBs similar to those observed during RSV infection, where N and P co-localize [18]. When co-expressed with P and N, M2-1 also localizes preferentially in these IBs, which has been linked to its interaction with P [19]. We thus analyzed the impact of the P mutations identified as critical for RdRp activity on the intracellular localization of M2-1 by fluorescence microscopy. Cells were cotransfected with expression vectors encoding P (WT and variants), N and M2-1-mCherry. All tested P variants were able to induce the formation of IBs (Fig 4). The M2-1-mCherry fusion protein accumulated in IBs in the presence of wild type (WT) P. M2-1 was also present in the IBs in the presence of P variant T108A, which did not impact transcription in the minigenome assay. In contrast, M2-1 was absent from the IBs for P variants that were defective for RdRp activity, F98A, L101A, Y102A, T105A, T105D, I106A, T108D and F109A, with the notable exception of F87A. It must be specified that mCherry fusion at the C-terminus of M2-1 does not affect the polymerase activity in the context of the minigenome, since ~ 70% of activity was recovered, as compared to WT M2-1 (S1 Fig). These results show that there is a good correlation between the presence of M2-1 in IBs and RdRp activity, except for F87A. They also point to the potential role of T108 phosphorylation for the presence of M2-1 in IBs.

### Effects of targeted P gene substitutions on the P–M2-1 interaction single out residue F87

**Fig 5:**
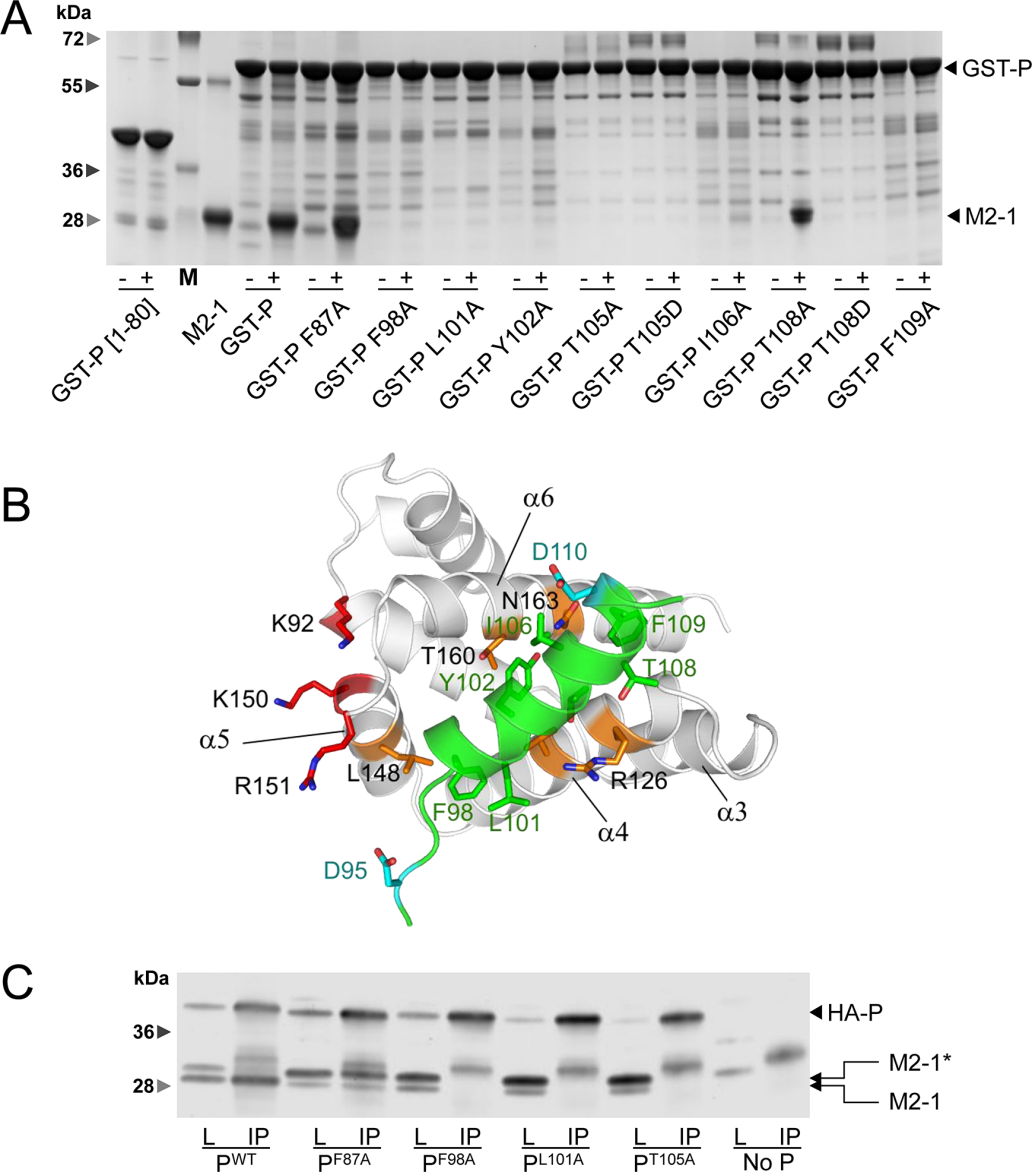
Effects of P substitutions on P–M2-1 interaction and M2-1 phosphorylation. (A) GST-P (WT and variants) and M2-1 with a C-terminal 6xHis-tag were expressed in *E*. *coli* and purified separately. Beads coated with GST-P (variants and WT), as well as GST-P[1–80] used as a negative control, were saturated with 3% BSA, and incubated alone (-) or in the presence of M2-1 (+), and washed. Complexes were resolved by SDS-PAGE and stained with Coomassie blue. M, molecular weight ladder. (B) Model of a P-M2-1 complex obtained by docking the P D95-F109 helix (green ribbon) onto a M2-1 protomer (grey ribbon, with indication of helix numbers). P residues critical for M2-1 binding are shown in sticks. M2-1 residues critical for P binding are in orange sticks, while residues involved in RNA-binding domain are in red sticks. (C) Analysis of the M2-1–P interaction in mammalian cells by immunoprecipitation. BSRT7/5 cells were transfected with plasmids encoding for N, HA-P (WT and variants) and M2-1. Immunoprecipitations (IP) from cell lysates (L) were performed 24 h post-transfection using an anti-HA antibody. P and M2-1 were revealed by Western blotting using anti-P and anti-M2-1 rabbit antisera. The star indicates the phosphorylated form of M2-1.

To determine whether point mutations affecting RSV transcription and M2-1 localization directly impact the P–M2-1 interaction, we performed GST pulldown assays using recombinant, non-phosphorylated GST-P and M2-1 proteins produced in *E*. *coli*. Fig 5A shows that substitutions F98A, L101A, Y102A, T105A, T105D, T108D and F109A were sufficient to impair P–M2-1 interaction *in vitro*, while M2-1 still interacted with the T108A and F87A variants.

We hypothesized that the M2-1 binding region in P, which displays helical propensity in solution folds into a stable helix upon M2-1—P complex formation, and we docked a structural model of the P helix (Fig 5B, green) onto the structure of M2-1_core_ under constraints obtained by mutagenesis and NMR interaction data. We have shown previously that the P binding site on M2-1 forms a groove between helices α4 and α6 and that the interaction surface on M2-1 is composed of hydrophobic residues (V127, L152, V156), neutral (N129, T130, S133) and basic residues (R126, R151) [19]. M2-1 R126D and L148A variants completely impaired P binding as well as its recruitment to cytoplasmic IBs. Critical P residues determined in the present study are rather hydrophic or neutral. However a 50% decrease in minigenome activity was observed for P variants D95A and D110A. These two acidic residues are at the edge of the P region involved in the interaction with M2-1 and could thus also play a role in this interaction (see Fig 5B). Altogether our observations strongly suggest that the binding surfaces of P and M2-1 involve both hydrophobic and electrostatic interactions.

To clarify the differences observed between F87A and the mutations affecting the P–M2-1 interaction, we performed immunoprecipitation after co-transfecting BSRT7 cells with plasmids encoding N, M2-1 and HA-P (WT and variants impairing *in vitro* transcription) and by precipitating with an anti-HA monoclonal antibody. The precipitated complexes were analyzed by Western blot using anti-P and anti-M2-1 polyclonal sera. As expected [29] [28], two main bands, corresponding to phosphorylated (upper band) and unphosphorylated (lower band) M2-1, were observed in cell extracts (Fig 5C). However, whereas M2-1 in the complex with WT P was mainly unphosphorylated, M2-1 was almost exclusively phosphorylated in the presence of all the P variants tested. Even more surprisingly, whereas only the unphosphorylated form of M2-1 co-precipitated with WT P, the F87A mutant was able to precipitate phosphorylated M2-1 as well. M2-1 remained phosphorylated with F98A, L101A and T105A P variants, which do not bind any form of M2-1, suggesting that the M2-1–P interaction is involved in M2-1 dephosphorylation. Taken together, these observations indicate that both unphosphorylated and phosphorylated forms of M2-1 can interact with P, and that the interaction between P and M2-1 favors M2-1 dephosphorylation. They also reveal that the F87A mutation impairs M2-1 dephosphorylation without impeding P–M2-1 binding.

### RSV P binds the cellular protein phosphatase 1 (PP1) by a "RVxF"-like motif

**Fig 6:**
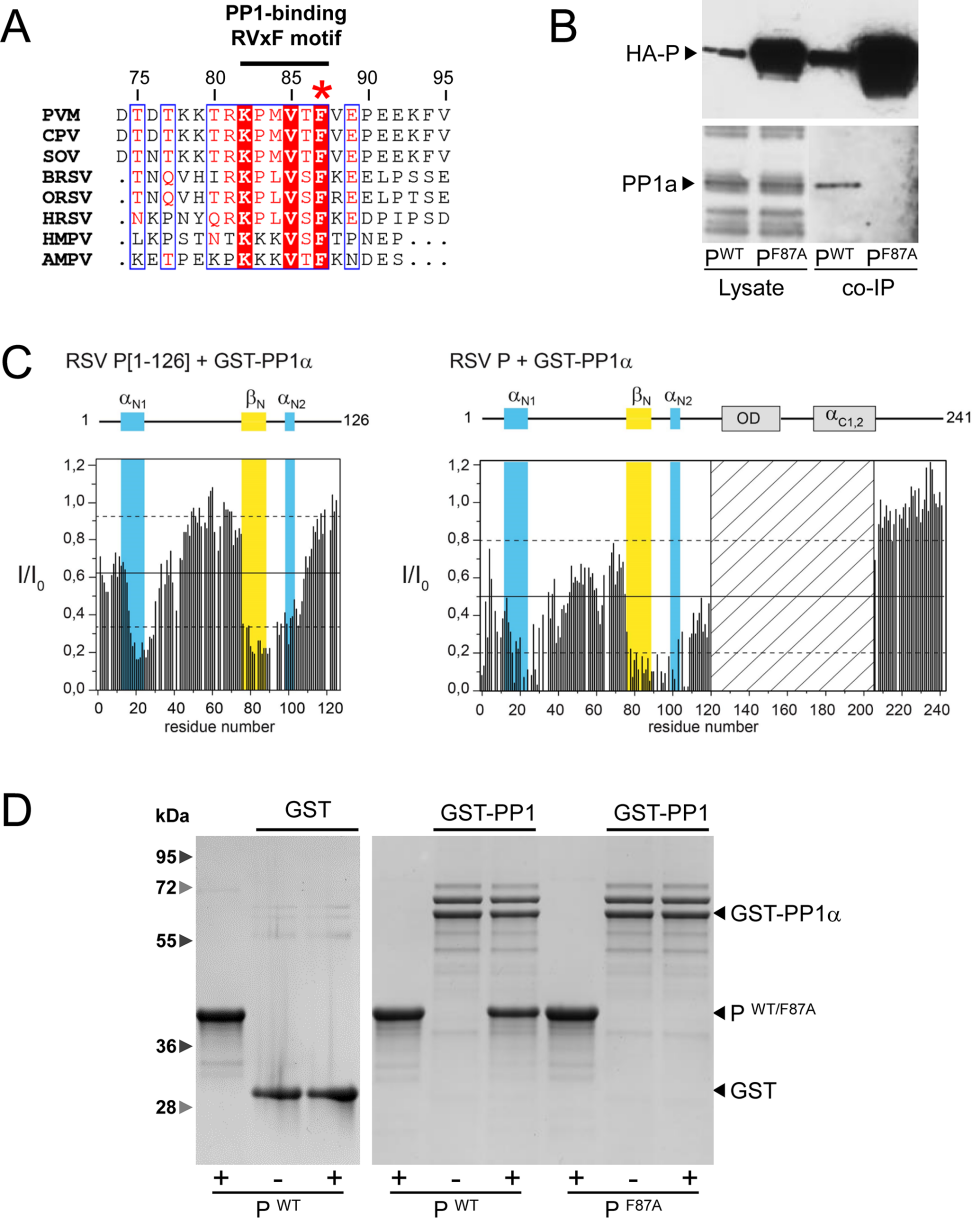
Phosphatase PP1 binds to the "RVxF" motif of RSV P. (A) Sequence alignment of *Pneumoviridae* P showing the conservation of the "RVxF" motif. Human RSV A strain (HRSV), bovine RSV (BRSV), ovine RSV (ORSV), pneumonia virus of mice (PVM), canine pneumovirus (CPV), swine orthopneumovirus (SOV), human metapneumovirus (HMPV) and avian metapneumovirus (AMPV) (accession codes AAX23990.1, NP_048051.1, Q83956.1, Q5MKM7.1, AHF88957.1, ANO40516.1, YP_012606.1, and AAF05910.1, respectively) P sequences were aligned by Clustal Omega and prepared with ESPript 3. Numbers correspond to RSV P amino acid residues. (B) BSRT7/5 cells were co-transfected with pN and pHA-P or p-HA-P[F87A]. HA-P (WT and variant) was immunoprecipitated from cell lysates and the presence of PP1 in the precipitate was analyzed by Western blot. L, cell lysate; IP, immunoprecipitated products. (C) NMR analysis of GST-PP1α binding to RSV P. Intensity ratios (I/I_0_), measured for each peak in ^1^H-^15^N BEST-TROSY spectra of 25 μM ^15^N-labeled P[1–126] or ^15^N-labeled full-length P, alone and in the presence of 2 molar equivalents of GST-PP1α, are represented in the bar diagrams. Straight and broken lines indicate mean and mean ± rmsd values. Regions with transient α-helical or β-sheet secondary structure [44] are highlighted by a colored background with the same color code as in Fig 1. The hatched area corresponds to a P region encompassing the OD and C-terminal helices for which peaks cannot be observed in the free form. (D) Proteins GST-PP1, M2-1, P WT and F87A mutant were expressed in *E*. *coli* and purified separately. Beads coated with GST or GST-PP1 were saturated with 3% BSA, and incubated with, P WT or F87A mutant and washed. Complexes were pulled down, resolved by SDS-PAGE and stained with Coomassie blue.

Since RSV P is required for M2-1 dephosphorylation, we hypothesized that P could associate with a cellular phosphatase, that in turn would be responsible for M2-1 dephosphorylation. For Ebola virus, which belongs to the *Filoviridae* family in the *Mononegavirales* order, it was recently shown that VP30, which shares functional and structural similarities with RSV M2-1, is dephosphorylated by PP1 [46], and that VP30 dephosphorylation is critical for viral transcription. PP1 does not recognize specific sequences on its target protein. Instead, substrate binding depends on its association with PP1-interacting proteins (PIPs) that function as targeting subunits [47]. A majority of known PIPs contain a short PP1-binding motif "RVxF" (R/K-K/R-x(0,1)-V/I-x-F/W/Y). RSV P contains a ^81^RKPLVSF^87^ sequence, which conforms to the "RVxF" motif. Sequence alignment of *Pneumoviridae* P shows high conservation of a "R/x-K-x-x-V-T/S-F" motif, which reduces to "R-K-P-x-V-T/S-F" for pneumoviruses (Fig 6A). RSV P thus harbors a degenerate "RVxF" motif containing the residue F87 and is most likely a PIP.

To determine whether P could associate with endogenous PP1, cells were co-transfected with pHA-P and p-N, and P was immunoprecipitated from cell lysates using an anti-HA antibody. The presence of cellular phosphatases was then analyzed by Western blot. The presence of PP1 was clearly revealed in the precipitated products using WT P (Fig 6B). With the P F87A variant, PP1 was no longer precipitated (Fig 6B), emphasizing the role of this residue. Of note, for unclear reasons, the F87A P variant was overexpressed as compared to WT P.

We then investigated whether RSV P could directly interact with PP1 by using NMR interaction experiments. We observed the perturbations in ^1^H-^15^N correlation spectra (BEST-TROSY) of ^15^N-labeled P[1–126] in the presence of unlabeled GST-PP1α. GST-PP1α induced line broadening of NMR signals, i.e. a decrease of intensity, notably in a region spanning residues K76-D90 (Fig 6C and S2 Fig). Control experiments in the presence of GST alone did not reveal any intensity perturbation (S2 Fig). The 76–90 region contains the degenerate "RVxF" motif identified above. It is adjacent to the M2-1 binding site, but was not perturbed by M2-1_core_ in P[1–126] (Fig 1C). The 76–90 region had already drawn our attention owing to more efficient ^15^N transverse relaxation than in adjacent IDRs and to its β-strand propensity in free P [44]. Since PPI RVxF" motifs adopt an extended β-strand conformation in complex with PP1, it was tempting to hypothesize that 76–90 region forms a primary PP1 binding site [48]. Similarly to M2-1_core_, GST-PP1α also perturbed the N^0^-binding region in P[1–126]. In contrast to M2-1_core_, when we performed interaction experiments with full-length P (Fig 6C and S2 Fig), perturbations in the vicinity of α_N1_ were still observed, so that we cannot rule out that they reflect direct binding to a second binding site [48]. The presence of GSH in the buffer did not affect intensities.

The ability of PP1 to interact specifically with P was thus investigated by GST pulldown using GST-PP1α and recombinant P, WT or F87A mutant, all produced in *E*. *coli*. As shown in Fig 6D, only WT P was efficiently pulled down by GST-PP1. In summary, these results demonstrate that the P–PP1 interaction is direct and that residue F87 of P plays a pivotal role in this interaction.

### RSV P recruits the cellular protein phosphatase 1 (PP1) to IBs, which is required for M2-1 accumulation in IBAGs

**Fig 7:**
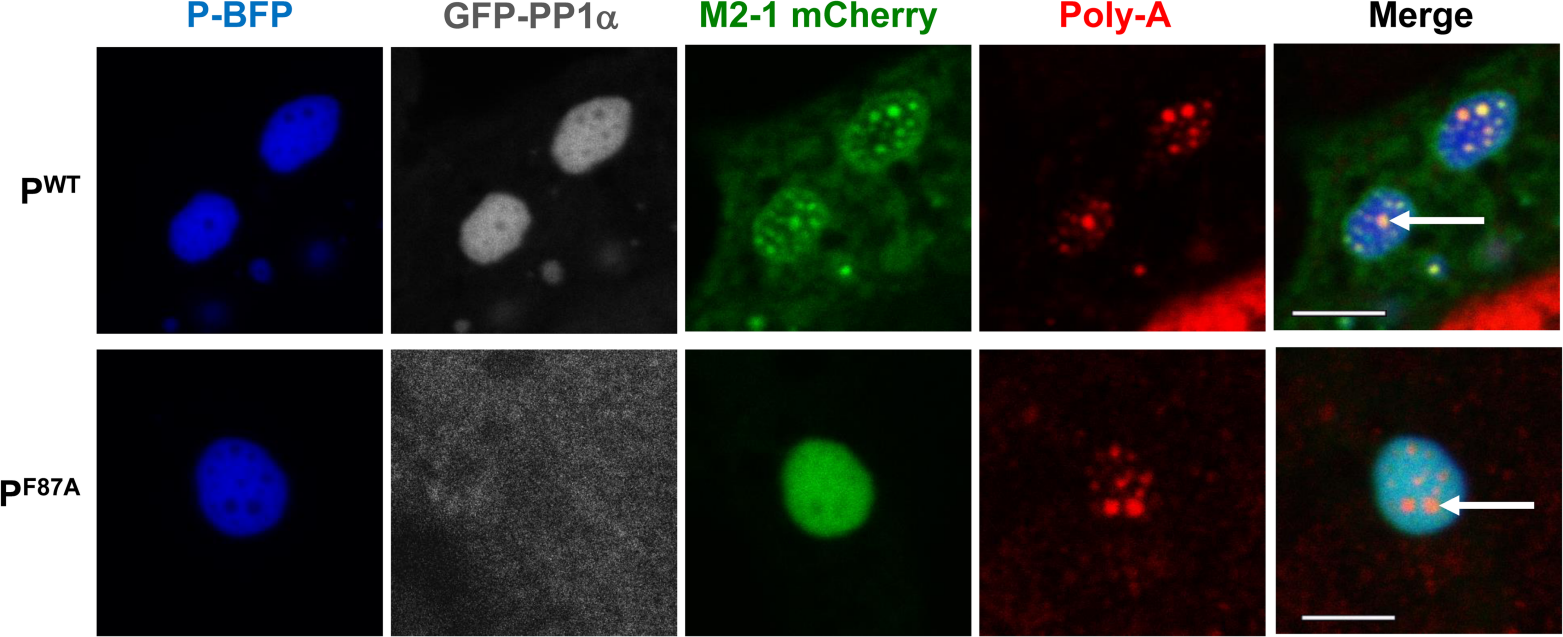
P-PP1 interaction allows recruitment of PP1 to IBs and M2-1 localization in IBAGs. BSRT7/5 cells were transfected with plasmids encoding the N, L and M2-1-mCherry proteins, the M/Luc RSV minigenome, and either wild type (WT) or F87A mutant P-BFP. Tagged proteins were expressed instead of the corresponding wild type as indicated on the pictures. FISH analyses were performed to detect poly(A) RNA (in red). The expressed tagged proteins are visualized thanks to their spontaneous fluorescence. IBs are delimited by the P fluorescence. Arrows point to IBAGs. Representative images from 3 independent experiments are shown. Images were taken under a Leica SP8 confocal microscope. Scale bars 5μm.

The presence of PP1 in IBs was analyzed by confocal microscopy. BSRT7/5 cells were co-transfected with plasmids expressing PP1-GFP, L, P-BFP, N, M2-1–mCherry proteins together with the pM/Luc vector expressing a firefly luciferase-reporter RSV minigenome, then fixed 24 hours post-transfection. Of note, BFP insertion between residues 73–74 of P, in a naturally disordered and poorly conserved region among *Pneumoviridae* [44], only moderately affected the polymerase activity in the context of the minigenome, since ~ 80% of activity was recovered, as compared to WT P (S1 Fig). As shown in Fig 7, PP1 fluorescence clearly overlapped with the WT P fluorescence, indicating that PP1 was significantly targeted to the IBs. In contrast, PP1 was no longer detected in IBs when expressed in the presence of the F87A P variant. These results revealed that RSV P recruits PP1 to IBs and that F87 plays a critical role in this process.

By studying the ultrastructure of IBs, we recently found that M2-1 colocalizes with viral neo-synthesized mRNA in IBAGs, revealed by a poly(dT) probe, and from which genomic viral RNA, N, P and L proteins are excluded [20]. We thus wondered if the absence of a P-PP1 complex could affect the localization of M2-1 in IBs. As the formation of IBAGs requires viral mRNA synthesis, we first verified that viral transcription can occur in the absence of M2-1 activity in our system. For that purpose, we engineered a new minigenome in which the first gene was replaced by the Gaussia luciferase coding sequence, upstream from the firefly luciferase gene. As shown in S3 Fig, whereas no Firefly luciferase activity was detected in the absence of M2-1, the Gaussia luciferase activity was still detectable although reduced to ~15%. This confirmed that M2-1 is not absolutely required for expression of the first gene but increases expression of this gene. Based on this result, transfected cells were prepared for FISH using poly(dT) probes and analyzed by confocal microscopy. Fig 7 shows that, in contrast to what was observed with WT P, in the presence of the F87A P mutant M2-1 was present throughout the IBs, where it colocalized with P, but absent from IBAGs as revealed by a poly(dT) probe. These results indicate that phosphorylated M2-1, which is still competent for P-binding, cannot associate with neo-synthesized viral poly-adenylated mRNAs.

**Fig 8:**
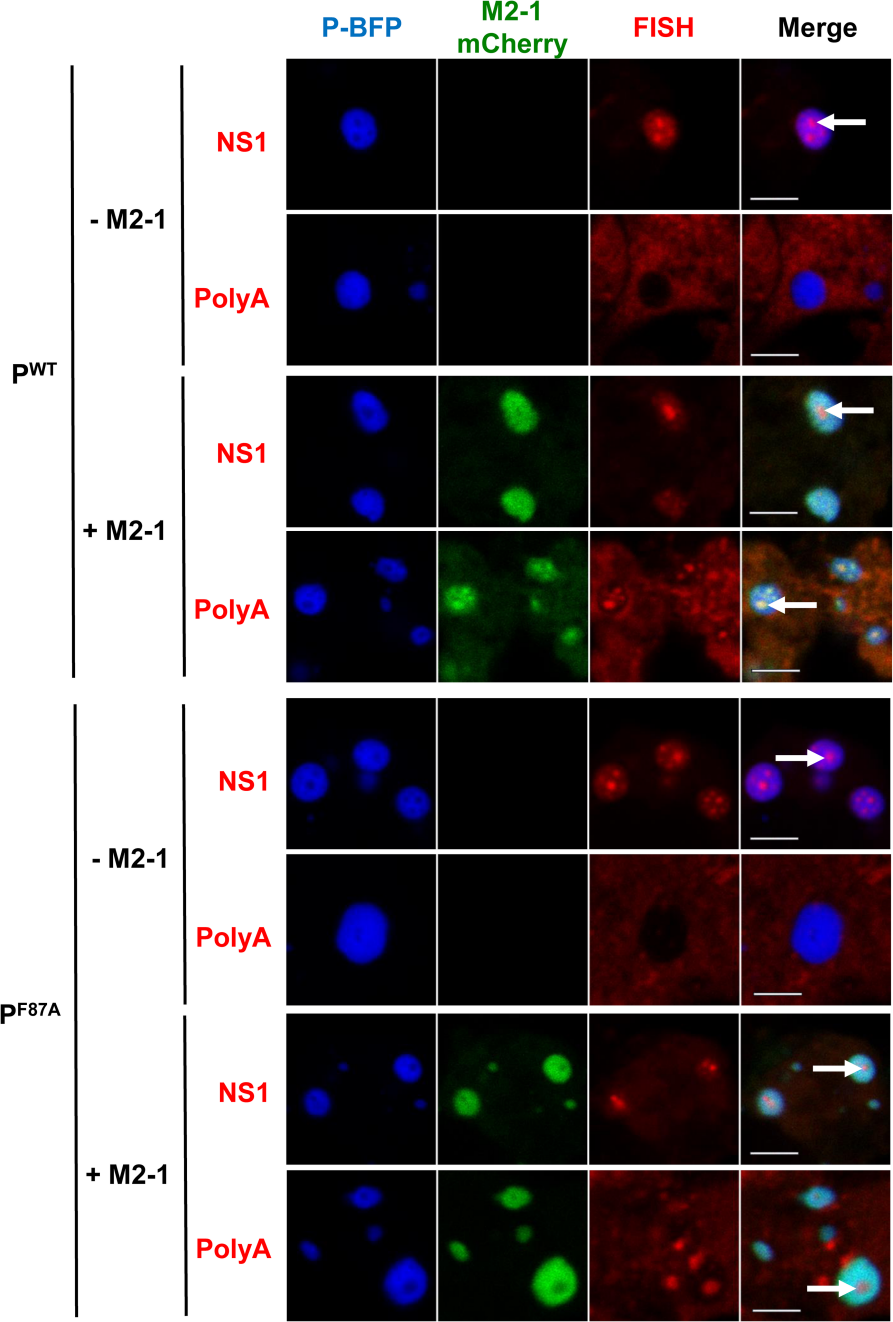
Effect of M2-1 on RSV mRNA poly-adenylation either in the presence or absence of PP1 in IBs. BSRT7/5 cells were transfected with plasmids encoding the N, L and M2-1-mCherry proteins, the M/Luc RSV minigenome, and either WT or F87A mutant P-BFP. FISH analyses were performed to detect poly(A) or NS1 RNAs (in red). The expressed tagged proteins are visualized thanks to their spontaneous fluorescence. IBs are delimited by the P fluorescence. Arrows point to IBAGs. Representative images from 3 independent experiments are shown. Images were taken under a Leica SP8 confocal microscope. Scale bars 5μm.

In a previous report we observed that IBAGs are not detected when using an oligo(dT) probe in the absence of M2-1 [20]. These results suggested that either M2-1 is needed for the formation of IBAGs, or that M2-1 binds to mRNAs and is carried to the IBAGs as a passenger, not as a required chaperone. To clarify this point, we used two different probes, an oligo(dT) and probes targeting the first transcription unit of the M/Luc minigenome (the NS1-M chimeric mRNA), and compared the presence of IBAGs in the absence or presence of either P WT or P F87A. Some pictures representing the trend of what we observed are shown in Fig 8; in the absence of M2-1, some IBAGs were detected when using NS1/M probes but not with an oligo(dT) probe. Similar results were observed with either WT or F87A P. Thus these results indicate that (i) transcription of the first gene can occur either in the absence of M2-1 or in presence of phosphorylated M2-1, as revealed by NS1 probes; (ii) poly-adenylation is affected in the absence of M2-1 but not in the presence of phosphorylated M2-1; and (iii) IBAGs revealed by either NS1 or oligo(dT) probes can form in the absence of M2-1 or in the presence of phosphorylated M2-1 which is excluded from them. In conclusion these results suggest that M2-1 is not essential for the formation of RNA aggregates calles IBAGs, and could be involved in mRNA poly-adenylation. They also suggest that a defect in M2-1 dephosphorylation does not affect mRNA poly-adenylation but binding of M2-1 to poly(A) RNAs after their synthesis.

### PP1 overexpression dephosphorylates M2-1 *in cellula*

**Fig 9:**
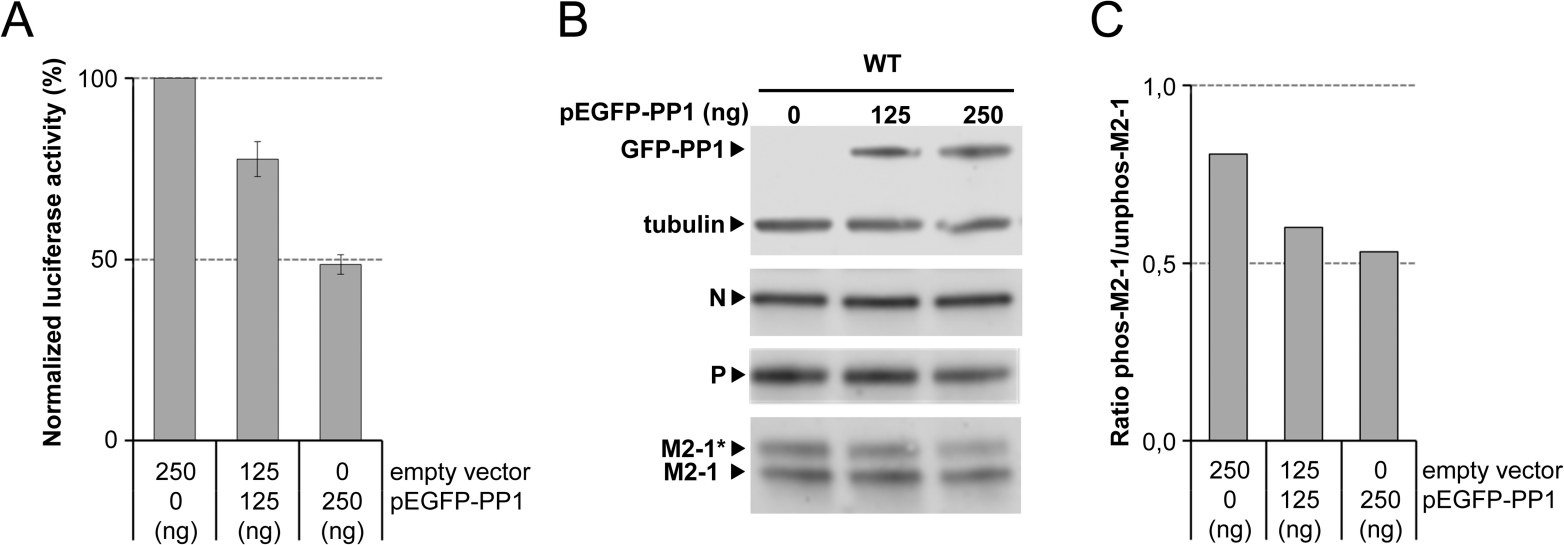
Effect of PP1 overexpression on RSV RNA polymerase activity and M2-1 phosphorylation. (A) Inhibition of RSV RNA polymerase activity by PP1 overexpression. BSRT7/5 cells were transfected with the RSV minigenome, and various amounts of pEGFP-PP1 vector. Viral RNA synthesis was quantified by measuring the Luc activity after cell lysis 24 h post-transfection. Each Luc activity value was normalized based on β-galactosidase expression and is the average of results from three independent experiments performed in triplicate. Error bars represent standard deviations. (B) Expression of phosphorylated (*) or unphosphorylated M2-1, N and P in BSR-T7/5 cells transfected with the RSV minigenome and with increasing quantities of pGFP-PP1vector. Cell extracts were resolved by SDS-PAGE 24 h post-transfection and analyzed by Western-blot using rabbit polyclonal anti-M2-1 or anti-P, and mouse monoclonal anti-α -tubulin antibodies. (C) Ratio of phosphorylated (Phos-M2-1) versus unphosphorylated (Unphos-M2-1) M2-1, normalized to levels of α-tubulin, in the presence of increasing amounts of pGFP-PP1vector. Signals were quantified using a Chemidoc Touch Imaging System (Bio-Rad, France).

A previous report suggested a requirement for cyclic phosphorylation/dephosphorylation of M2-1 for efficient antitermination function [24]. To further demonstrate that PP1 is involved in M2-1 dephosphorylation *in cellula*, PP1 was overexpressed in BSRT7/5 cells in the context of the RSV minigenome. Fig 9A shows that RSV RdRp activity, as revealed by Luc activity, significantly decreased in a dose-dependent manner with pEGFP-PP1 plasmid addition, while P and N expression levels were not affected (Fig 9B). However, although the expression level of unphosphorylated M2-1 was not or poorly affected (Fig 9B), overexpression of PP1 significantly induced a decrease in phosphorylated M2-1, and thus a decrease of the ratio of phosphorylated versus unphosphorylated M2-1 (Fig 9B and 9C), confirming that PP1 is involved in M2-1 dephosphorylation in living cells.

## Discussion

### RSV P recruits M2-1 to IBs

**Fig 10:**
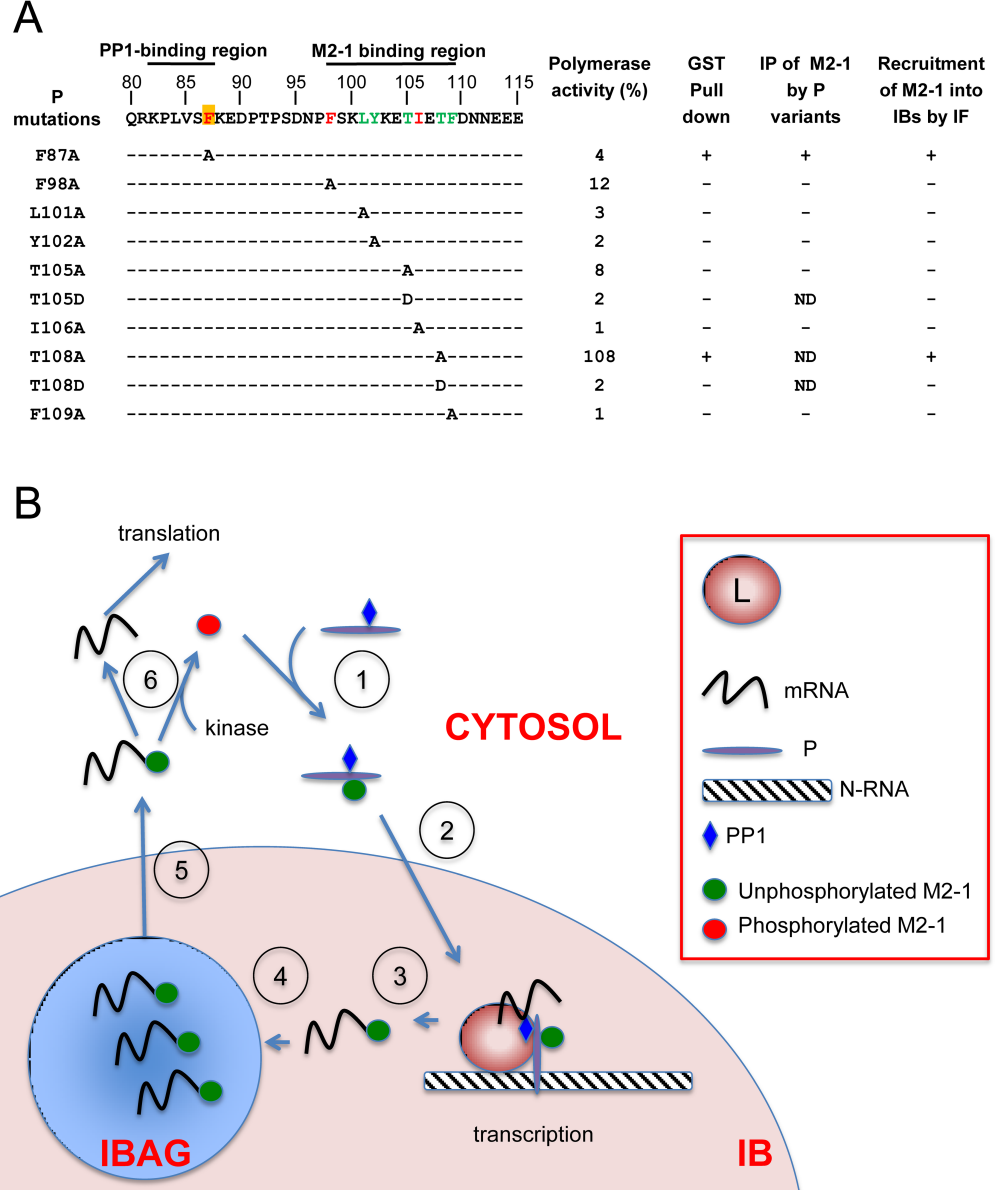
Model for phosphorylation turnover of M2-1 taking into account the P–M2-1 and P–PP1 interactions. (A) The primary sequence of the 80–115 region of P is indicated at the top. Amino acid residues previously identified as critical for M2-1 binding [33, 34] are in green, newly identified residues F98 and I106 are in red. Residue F87 critical for M2-1 dephosphorylation and efficient transcription is highlighted in red font on a yellow background. The left-hand column shows amino acid substitutions. Right-hand columns summarize the impact of P mutations on (i) the polymerase activity, (ii) *in vitro* interaction between P and M2-1 using recombinant proteins, (iii) interaction between P and M2-1 assessed by co-immunoprecipitation from transfected BSRT7/5 cells, and (iv) on the recruitment of M2-1 to IBs; ND, not determined. (B) Diagram of M2-1 phosphorylation turnover. (1) M2-1 is phosphorylated (at Ser 58 and 61), binds to P in the cytosol and is dephosphorylated by PP1 in the P–PP1 complex. (2) The P–PP1–M2-1 complex is directed to IBs (pink) where transcription takes place. (3) M2-1 binds to neo-synthesized mRNAs at the end of transcription and (4) M2-1-mRNAs complexes are concentrated in IBAGs (blue) before being released in the cytosol (5). M2-1 is then phosphorylated by a cellular kinase and parts with viral mRNAs which will be used for translation of viral proteins (6).

The efficient activity of the RSV RdRp complex depends on regulated and highly specific protein-protein interactions, which are potential targets for antiviral therapy [49]. P plays a pivotal role through multiple interactions with L, N and M2-1, mediated by its high structural plasticity linked to its disordered regions [44, 50]. The M2-1 binding region has been mapped previously to P residues 100–120, using proteins from two RSV strains (A2 and Long), and the role of specific residues L101, Y102, F109, T105 and T108 was highlighted [33], [34]. By combining NMR, biochemical and functional approaches based on an RSV minigenome and microscopy, we precisely mapped the M2-1 binding region of P to a stretch encompassing residues 93–110. We identified 7 residues of P that are directly involved in this interaction, summarized in Fig 10A. These include two newly identified residues F98 and I106 and validate the major role of hydrophobic P residues in the P–M2-1 interaction [33].

Furthermore, we found a perfect correlation between the capacity of P to interact with M2-1, RdRp activity and the presence of M2-1 in IBs. These results indicate that M2-1 cannot reach IBs without P, P acting as a recruiter for M2-1, and are in agreement with previous observations obtained with M2-1 variants unable to interact with P [19].

### Dephosphorylation of M2-1 is mediated by a P-PP1 complex

M2-1 is mainly unphosphorylated in the context of an RSV infection, whereas the phosphorylated state is predominant when M2-1 is expressed alone in cells, S58 and S61 being the main sites of phosphorylation [26]. Previous experiments showed that phosphoablatant substitutions S58A/S61A as well as phosphomimetic substitutions S58D/S61D reduced RSV transcription activity to less than 20% and to ∼40% as compared to WT M2-1, respectively [24, 26]. These data suggest that a cyclic turnover of phosphorylation-dephosphorylation of M2-1 is required for efficient RSV transcription.

Here we observed that unphosphorylated M2-1 was predominant in cell lysates when co-expressed with WT P. A reverse situation was observed when M2-1 was co-expressed with P variants F98A, L101A, T105A that do not interact with M2-1, M2-1 being mainly phosphorylated, suggesting that P binding induces M2-1 dephosphorylation (Fig 5C). The finding that M2-1 is predominantly phosphorylated in the presence of another P variant F87A, which efficiently pulled down M2-1, confirmed that P is capable of interacting with both phosphorylated and unphosphorylated forms of M2-1 [23]. Altogether these results indicated that (i) P mediates M2-1 dephosphorylation or prevents M2-1 phosphorylation and that (ii) a P region adjacent to the M2-1 binding region comes into play for this activity.

Previous studies suggested that RSV P could be a target of cellular phosphatases PP1 and PP2A in cultured cells [38, 51]. PP1 is a well-characterized and conserved Ser/Thr phosphatase holoenzyme. It is composed of a variable regulatory subunit that determines the localization, activity, and substrate specificity of the phosphatase and of one of three highly homologous catalytic phosphatase subunits PP1α, PP1γ, or PP1β/δ (reviewed in [47, 48]). PP1 is the most widely expressed and abundant Ser/Thr phosphatase and is estimated to catalyze about one third of all protein dephosphorylations in eukaryotic cells. It dephosphorylates hundreds of key biological targets by associating with nearly 200 regulatory proteins to form highly specific holoenzymes. Of note, many of the > 200 established PIPs are predicted to be intrinsically disordered like RSV P.

The defect in M2-1 dephosphorylation observed for the P F87A variant mainly argued in favor of the hypothesis of the recruitment by P of the PP1 cellular phosphatase through a RVxF-like motif, upstream of the M2-1 binding site. This hypothesis was consolidated by several complementary approaches showing that (i) WT P, but not the F87A variant, could bind PP1 *in vitro* and *in cellula;* (ii) PP1 colocalized with WT P, but not with the F87A variant, in IBs; and (iii) overexpression of PP1 increased the unphosphorylated/phosphorylated M2-1 ratio. Altogether, our data show that RSV P can be considered as a PP1-interacting protein (PIP), targeting PP1 to the M2-1 substrate.

### Mode of action of PP1 for M2-1 dephosphorylation

The degenerate "RVxF" motif in RSV P, ^81^RKPLVSF^87^, is well conserved among *Pneumoviridae*, with a consensus KxxVxF (Fig 6A). The lysine residue is surrounded by basic residues, with an arginine just upstream for orthopneumoviruses, and two lysines downstream for metapneumoviruses. Thus, it is likely that the P proteins of *Pneumoviridae* share the property of interacting with PP1 to regulate viral protein phosphorylation. Notably, M2-1 protein is unique to *Pneumoviridae* and present in all members of this virus family. It was shown that residues S57 and S60 of hMPV M2-1 protein, which are equivalent to RSV M2-1 S58 and S61, are also critical for virus replication, consistent with the critical role of cyclic phosphorylation/dephosphorylation of hMPV M2-1 for efficient RNA synthesis [52]. It is thus expected that hMPV and RSV present the same mechanism of regulation of M2-1 dephosphorylation.

It is noteworthy that PP1 was previously shown to play a significant role in the replication of several viruses, including papovavirus, adenovirus, human immunodeficiency virus 1 (HIV-1) and 2, Ebola virus (EBOV), and Rift Valley Fever virus [46, 53, 54]. Within *Mononegavirales*, the VP30 protein from filoviruses is the only protein that shares structural and functional similarity with the M2-1 protein from *Pneumoviridae*. It was shown that the phosphorylation status of VP30 was also regulated by PP1 [46]. It has also been demonstrated that PP1 regulates the innate immune responses for numerous RNA viruses, such as influenza virus, Sendai virus, dengue virus, and picornavirus [55]. The V protein of measles virus was shown to interact directly with PP1α/γ, via a canonical PP1-binding motif, ^288^RIWY^291^, preventing PP1-mediated dephosphorylation of MDA5, a cytosolic sensor crucial for innate immune defense against various RNA viruses, thereby impairing its activation [56].

### Potential role of cyclic M2-1 phosphorylation/dephosphorylation

Previous reports indicated that the phosphorylation state of M2-1 regulates the affinities with its partners [25]. The highest affinities of RSV M2-1 (produced as recombinant protein in *E*. *coli*, i.e. unphosphorylated) for RNA were found with poly(A) (Kd~20 nM) and sequences present on viral mRNAs that are complementary to GE signals (Kd ~ 46 nM) [19, 24]. We previously determined that the phosphomimetic S58D/S61D substitution decreased the RNA binding affinity as compared to WT M2-1 [24]. This is consistent with the crystal structure of full-length tetrameric RSV M2-1 [24], where S58 and S61 are located on a flexible loop, facing the RNA binding region located in M2-1_core_. Addition of a negative charge on these residues by phosphorylation could thus affect the interaction with RNA. Recombinant tetrameric P and M2-1 proteins (produced in *E*. *coli*, i.e. unphosphorylated) form a complex with a 1:1 stoichiometry and a Kd of ~ 8.1 nM [32]. The affinities of P and RNA for M2-1 are therefore comparable, which would be in line with a switch of M2-1 between the two M2-1–RNA and M2-1–P complexes.

This assumption was confirmed by comparing the organization of IBs in the presence of WT or F87A P variant in our minigenome assay. We previously observed a distinct compartment in IBs that contains concentrated viral mRNAs and M2-1 (IBAG). The rest of the IBs holds all the other proteins of the RSV polymerase complex together with the genomic RNA [20]. When the P F87A variant was used, PP1 was absent from IBs, and M2-1, which was found mainly in a phosphorylated form (see Fig 5C), was no longer associated with IBAGs. The M/Luc minigenome we used for fluorescence microscopy studies has two transcription units (see materials and Methods); the first one can be transcribed in the absence of M2-1, although with a 5 to 7 fold reduction (see S3 Fig), while the second one coding for Firefly luciferase fully depends on a functional M2-1 [17, 23, 57]. It is thus likely that the poly(A) RNA seen in IBs when P F87A was used represents transcription from the first gene. These results also indicate that IBAGs form in the absence of dephosphorylated, competent for mRNA binding, M2-1. Finally, by using two different RNA probes, i.e. NS1 and poly(dT), we observed different effects on viral RNA poly-adenylation when M2-1 was either absent or co-expressed with P F87A and thus in the absence of PP1; in the first case some residual transcription was detected with the NS1 probe but not with the poly(dT) probe; in the second case (phosphorylated M2-1 only), both probes could detect some IBAGs. Together these results suggest that (i) poly-adenylation of the first transcription unit is impaired in the absence of M2-1 and (ii) phosphorylated M2-1 can help poly-adenylation, although it is excluded from IBAGs after transcription.

To summarize, RSV P can be considered as a newly identified PIP, targeting PP1 to the M2-1 substrate. To our knowledge, this is the first report showing such a mechanism of regulation of *Pneumoviridae* transcription. Recently, we have shown that, in RSV-infected cells, viral RNA synthesis occur in IBs, M2-1 and viral mRNAs concentrating in IB sub-compartments called IBAGs [20]. The data indicated that IBAGs are dynamic structures, allowing the sorting of viral mRNAs and their transport to the cytoplasm together with M2-1. Accordingly, we propose the following model (Fig 10B): in the cytoplasm of infected cells the P protein binds PP1 and the phosphorylated form of M2-1 allowing their recruitment into IBs. M2-1 works as an anti-terminator of transcription in IBs and could intervene as a poly-adenylation factor. Once dephosphorylated by PP1, M2-1 has a higher affinity for RNA, in particular for GE and poly(A) sequences at the end of the transcripts. Terminated mRNAs concentrate in IBAGs and drag M2-1 along. M2-1 could act in a manner similar to the cellular poly(A) binding protein, protecting mRNA from degradation and perhaps playing a role in the transport of mRNAs from IBAGs to the cytosol in order to translate viral mRNAs, and possibly playing an active role in translation. M2-1 phosphorylation could then occur in the cytosol, resulting in the detachment of M2-1 from the poly(A) tail of mRNA, before being recycled by P and redirected to IBs for a new round of transcription.

## Materials and methods

### Plasmid constructs

All viral sequences were derived from the human RSV strain Long. The full length or segments of the P gene were PCR amplified by using *Pfu* DNA polymerase (Stratagene, Les Ulis, France) and cloned into pGEX-4T-3 bacterial expression vector (GE Healthcare) at BamHI-SmaI sites to engineer the pGEX-P and derived plasmids. The M2-1 cDNA [23] was subcloned into pET-22b+ (Novagen) to allow bacterial expression of full-length M2-1 with a C-terminal poly-histidine tag. Plasmids for eukaryotic expression of the human RSV (Long strain) proteins N, P, M2-1, and L, designated pN, pP, pM2-1, and pL, respectively, and pM/Luc have been described previously [22, 23]. The pM/Luc minigenome has two transcription units; the first ORF is a chimera between NS1 (327 first nucleotides) and M genes (138 last nucleotides), and transcription termination depends on a N Gene End sequence. The second ORF codes for firefly luciferase and ends with a SH Gene End sequence The first and second mRNA expressed by this minigenome are 698 and 1897 nucleotides in length, excluding the poly(A) tail, respectively. A second minigenome containing Gaussia and Firefly luciferases was engineered and synthesized by Genscript. This pGaussia/Firefly minigenome is similar to the pM/Luc minigenome excepted that it contains the Gaussia luciferase gene upstream in place of the NS1-M chimeric gene (complete sequences available on demand). Point mutations were introduced in the P sequence by site-directed mutagenesis using the QuikChange site-directed mutagenesis kit (Stratagene). To generate the plasmid pHA-P, complementary oligonucleotides encoding a hemagglutinin (HA) tag epitope (MYPYDVPDYA) were annealed and inserted at the BamHI restriction site in frame at the 5’ end of the P gene in pP. For the eukaryotic expression of a M2-1–mCherry fusion protein, the mCherry gene was amplified by PCR from the pmCherry vector (Clontech) and cloned in frame at the 3’ end of the M2-1 gene at BglII-XhoI sites in pM2-1. To engineer the pP-BFP vector, first a NheI site was introduced at nucleotides 741–746 of the P sequence; then, the BFP gene was PCR amplified from the pTagBFP-Tubulin vector (Evrogen) and inserted at the NheI restriction site, between P residues 73–74. The eukaryotic expression vector pEGFP(C1)-PP1α was purchased from Addgene. The PP1A insert (residues 7–300) was subcloned into pGEX-4T-3 at BamHI-XhoI sites. Sequence analysis was carried out to check the integrity of all the constructs. All the oligonucleotide sequences are available on request.

### Bacterial expression of recombinant proteins and purification

For M2-1 expression, *E*. *coli* BL21(DE3) (Novagen) bacteria were transformed with pET-M2-1 plasmid, and bacteria were grown at 37°C for 8 h in Luria-Bertani medium (LB) containing 100 μg/ml ampicillin. Protein expression was induced by adding one volume of fresh LB medium, 400 μM isopropyl β-D-1-thiogalactopyranoside (IPTG) and 50 μM ZnSO_4_ for 16 h at 28°C. Cultures were centrifuged at 5,000 *g* for 15 min and the pellet was resuspended in lysis buffer (20 mM Tris-HCl pH 7.4, 150 mM NaCl, 50 mM imidazole, 0.1% Triton X-100 and 1 mg/ml lysozyme). Benzonase (Novagen) was then added to the lysates (final concentration 5U/mL), which were further incubated for 1h at room temperature under rotation. NaCl was then added to reach a final concentration of 1M and lysates were clarified by centrifugation at 10,000 *g* for 1h at 4°C. The supernatant was loaded onto a 5-mL HiTrap IMAC column (GE Healthcare) charged with 0.2 M ZnSO_4_ and equilibrated with low-imidazole buffer and high salted buffer (50 mM imidazole, 20 mM Tris-HCl [pH 7.4], 1M NaCl) using a 50-mL Superloop. Then a linear gradient of 1–0.15M NaCl was applied to reduce the concentration in NaCl. Finally, a linear gradient of 80–800 mM imidazole in the same buffer was applied to elute M2-1 fractions containing the His-tagged proteins. After equilibration with 20 mM Tris-HCl [pH 7.4], 150 mM NaCl buffer, M2-1 was further purified by size exclusion chromatography on a HiLoad Superdex-200 column with a 120-mL total bed volume (GE Healthcare). Appropriate fractions were pooled. M2-1 protein was confirmed RNA-free by spectrophotometry (OD 260/280 ratio) and stored at 4°C.

For P expression, *E*. *coli* BL21(DE3) bacteria transformed with pGEX-P derived plasmids were grown as described above. Bacterial pellets were resuspended in lysis buffer (20 mM Tris/HCl pH 7.4, 60 mM NaCl, 1 mM EDTA, 1 mg/mL lysozyme, 1 mM DTT, 0,1% Triton X-100) supplemented with complete protease inhibitor cocktail (Roche) for 1 h on ice. Benzonase was then added and the lysate was incubated for 1 h at ambient temperature under rotation. The lysates were centrifuged at 4°C for 30 min at 10,000 *g*. Glutathione-Sepharose 4B beads (GE Healthcare) were added to the clarified supernatants and the mixtures were incubated overnight at 4°C under rotation. The beads were washed with lysis buffer, three times with 1X PBS and then stored at 4°C in an equal volume of PBS.

For PP1 expression, chaperone competent cells pGro7/BL21 (Takara) were transformed with pGEX-PP1 and grown as previously described [58]. Briefly, an overnight starter culture was grown at 37°C in LB medium supplemented with antibiotics and 1 mM MnCl_2_. PP1 production was initiated by inoculating 1/2 liter of LB supplemented with 1 mM MnCl_2_ with 35 ml of starter culture. The bacteria were grown at 30°C to an OD of ∼0.5, then arabinose was added (2 g/l) to induce the expression of the GroEL/GroES chaperone. When OD was ∼1, the temperature was lowered to 10°C (ice-bath) and the expression of PP1 was induced with 0.1 mM IPTG for ∼20 h. Culture was centrifuged at 5,000 *g* for 15 min and bacterial pellets were resuspended in lysis buffer (20 mM Tris/HCl pH 8.0, 150 mM NaCl, 1 mM MnCl_2_, 1 mg/mL lysozyme, 1 mM DTT, 0,1% Triton X-100) supplemented with complete protease inhibitor cocktail (Roche) for 1 h on ice and treated as described above except that NaCl was then added to reach a final concentration of 700mM.

For NMR experiments ^15^N-labeled P and two N-terminal fragments of P, P[1–126] (residues 1–126) and P[1–163] (residues 1–163), were produced in M9 medium supplemented with ^15^N-labeled NH_4_Cl (Eurisotop) and glucose and purified following the same protocol as for full-length P. The core domain of M2-1 (residues 58–177) was prepared as described previously [19]. Proteins were cleaved from GST with biotinylated thrombin (Novagen). Thrombin was later removed using streptavidin resin (Novagen). The samples were subsequently concentrated on centrifugation filter units (Amicon Ultra) to 50–150 μM and dialyzed against NMR buffer (20 mM sodium phosphate pH 6.8, 100 mM NaCl). GST-PP1α was eluted from GSH-sepharose beads by using 50 mM glutathione, concentrated and dialyzed against NMR buffer. Purity was assessed by SDS-PAGE and by mass spectrometry.

### GST pulldown assays

GST pull-downs were performed by incubating 50μl of a 50% slurry of Glutathione-Sepharose 4B beads (GE Healthcare) containing either GST-PP1 or GST-P (WT and mutants) at 25 μM in 20mM Tris/HCl [pH 7.4], 150mM NaCl (TN buffer) with a 3-fold molar excess of M2-1 or P and BSA 3%. After 1 h at 20°C under agitation, the beads were washed extensively with TN buffer, boiled in 25 μl of Laemmli buffer and analyzed by SDS-PAGE and Coomassie blue staining.

### Cell culture

BSRT7/5 [59] cells were maintained in Eagle’s minimum essential medium and Dulbecco’s modified Eagle’s medium, respectively, supplemented with 10% fetal calf serum, 2 mM L-glutamine, and penicillin–streptomycin solution. The cells were grown at 37°C in 5% CO2. Cytotoxicity measurements were performed using the CellTiter-Glo Luminescent cell viability assay (Promega).

### Minigenome experiments

BSRT7/5 cells at 90% confluence in 24-well dishes were transfected using Lipofectamine 2000 (Invitrogen) with a plasmid mixture containing 0.25 μg of pM/Luc minigenome, 0.25 μg of pN, 0.25 μg of pP (WT and mutants), 0.125 μg of pL, and 0.06 μg of pM2-1, as well as 0.06 μg of pSV-β-Gal (Promega) to normalize transfection efficiencies as previously described [23]. Transfections were done in triplicate and each independent transfection was performed three times. Cells were harvested at 24 h post-transfection and lysed in luciferase lysis buffer (30 mM Tris [pH 7.9], 10 mM MgCl_2_, 1 mM dithiothreitol [DTT], 1% [vol/vol] Triton X-100, and 15% [vol/vol] glycerol). Luciferase activities were measured for each cell lysate after injection of lysis buffer supplemented with ATP and D-luciferin (final concentrations 1mM each) with an Infinite 200 Pro (Tecan, Männedorf, Switzerland) and normalized to β-Gal expression levels.

### Fluorescence microscopy

BSRT7/5 cells grown on coverslips were transfected with pN, pM2-1–mCherry and pP or p-BFP (WT or variants) using Lipofectamine2000 (Invitrogen). At 24 h post-transfection, samples were fixed in 4% paraformaldehyde (PFA) for 30 min, and permeabilized in PBS containing 0.1% Triton X-100 and 3% BSA. Coverslips were incubated for 1h at room temperature with primary antibodies, washed, and then incubated for an additional hour with Alexa Fluor 488 goat anti-mouse IgG (Invitrogen). For P detection the mouse anti-P monoclonal antibody 021/2P [18] was used. Coverslips were mounted with ProLong Gold Antifade reagent containing DAPI (Life Technologies). Cells were observed with a Nikon TE200 inverted microscope equipped with a Photometrics CoolSNAP ES2 camera. Images were processed using MetaVue software (Molecular Devices).

Confocal microscopy was used to study IB ultrastructure. Z-stack image acquisitions of multi-labeled (P-BFP, N, M2-1-Cherry, FISH) cells were performed under a 63x apochromatic lens and a numerical zoom comprised between 1x and 15x (LSAF acquisition software) under the WLL Leica SP8 microscope and representative pictures were taken.

### Fluorescent in situ hybridization (FISH)

Infected cells were fixed 24 h p.i. and permeabilized as described above. Endogenous biotin was blocked in PBS-BSA 1% (w/v) supplemented with free streptavidin (4 μg/ml) for 1h. Coverslips were rinsed 3 times with PBS, post-fixed 10 min at 4°C in formaldehyde 4% (v/v), rinsed 2 times with PBS, and incubated in hybridization mix (2x SSC (1x SSC is 150 mM NaCl and 15 mM sodium citrate), dextran 10% (w/v), formamide 20% or 50% (v/v) for oligo(dT) and NS1 probes, respectively, 1 mg/ml herring sperm DNA; mRNAs were detected by using either 3’-biotinylated poly(dT) or a pool of NS1 oligonucleotides (for sequences see ref. [20] at a final concentration of 100 μM in a humidified chamber at 37°C for 3h. Next, cells were washed 2 times at 42°C with the following 3 solutions: 2x SSC plus formamide 20% (v/v), 2x SSC, 1x SSC and finally PBS at room temperature. Probes were then detected by incubating cells with streptavidin-Alexa Fluor 647 conjugate (8 μg/ml) in PBS-BSA 1% (w/v) during 1h prior to 3 washes with PBS. Cells were then submitted to immunofluorescent staining and confocal microscopy.

### Immunoblotting

Cells were lysed for 30 min at 4°C in lysis buffer (20 mM Tris [pH 7.4], 150 mM NaCl, 0.1% Triton X-100) supplemented with a complete protease inhibitor cocktail (Roche). Cell lysates were spun for 10 min at 10,000 *g*; supernatants were recovered, mixed with Laemmli buffer, and boiled. Proteins were resolved by SDS-PAGE and transferred onto nitrocellulose membranes. The membranes were incubated in blocking solution (1X PBS, 0.05% Tween 20 supplemented with 5% milk) for 1 h. Blots were incubated with primary antibodies in blocking solution: rabbit anti-P and anti-M2-1 antisera [31], mouse monoclonal anti-α-tubulin antibody (Sigma), rabbit polyclonal anti-PP1A antibody (Abcam) and rat monoclonal anti-HA-peroxidase antibody (Roche). The membranes were rinsed with PBS containing 0.05% Tween 20 and incubated for 1 h with the appropriate HRP-conjugated secondary antibodies diluted in blocking solution. The membranes were rinsed, and immunodetection was performed by using an enhanced chemiluminescence (ECL) substrate (BioRad, France).

### Coimmunoprecipitation assays

BSRT7/5 cells were cotransfected with pHA-P (WT and variants), pN and pM2-1. After 24 h, transfected cells were lysed for 30 min at 4°C in ice-cold lysis buffer (20 mM Tris HCl [pH 7.4], 150 mM NaCl, 0.1% Triton X100, 20μM RNAse A and 15% glycerol) with a complete protease inhibitor cocktail (Roche). Cell lysates were centrifuged at 4°C for 10 min at 10,000 *g* and incubated overnight at 4°C with a rat anti-HA monoclonal antibody (Roche cl. 3F10) coupled to magnetic beads (Invitrogen). The beads were then washed 3 times with lysis buffer and 1 time with PBS, proteins were boiled in Laemmli buffer for 5 min and samples were subjected to SDS-PAGE and immunoblotting as described above.

### NMR spectroscopy

^1^H-^15^N correlation spectra, Heteronuclear Single Quantum Correlation (HSQC) or BEST-TROSY, were measured at a temperature of 288 K on Bruker Avance III 800 or 950 MHz spectrometers equipped with cryogenic TCI probes. 7% D_2_O was added to the samples to lock the magnetic field. Spectra were processed with Topspin 3.2 (Bruker Biospin) and analyzed with CCPNMR 2.2 software [60].

### Modelling

Flexible docking was carried out on the guru interface of the Haddock Webserver [61, 62], using the X-ray structure of M2-1_core_ extracted from PDB 4C3E, chain B. Models of a P helix spanning residues 94–112 were built using CYANA 3.2 [63] under torsion angle constraints obtained from backbone chemical shifts of P. Following active M2-1 (126, 127, 129, 130, 133, 148, 152, 156, 160, 163) and P residues (98, 101, 102, 105, 106, 108 and 109) were identified by mutagenesis experiments or by NMR interaction experiments, in this work or in [19]. Passive residues were automatically defined around active residues. 1000 initial structures were generated. 200 final structures were refined in water and clustered according to RMSD criterion. More than 50% structures clustered in the same cluster with the best Haddock score. Statistics are shown in S1 Table.

## Supporting information

S1 Fig Effects of M2-1-mCherry and P-BFP for RSV polymerase activity. BSRT7/5 were transfected with pMT/Luc, pP or p-P-BFP, pL, pN and either pM2-1 or pM2-1-cherry, and Luc reporter activity was measured.
(DOCX)

S2 Fig Perturbations of GST-PP1α in NMR spectra of P. (Upper panel) Superimposed ^1^H-^15^N BEST-TROSY spectra of 25 μM ^15^N-labeled P[1–126] or ^15^N-labeled P, alone (magenta contours) and in the presence of 2 molar equivalents of GST-PP1α (black contours) are shown with residue-specific assignments. (Lower panel) Superimposed ^1^H-^15^N HSQC spectra of 10 μM ^15^N-labeled P[1–126], alone (green contours) and in the presence of 10 molar equivalents of GST (black contours). The buffer of the P[1–126]+GST sample contained 25 mM glutathione that yields natural abundance ^15^N signals indicated by stars.
(DOCX)

S3 Fig Comparative normalized activities of Gaussia and Firefly luciferase in the absence (-) or presence (+) of M2-1. BSRT7/5 were transfected with pGaussia/Firefly minigenome vector, pP pL, pN and either pM2-1 or an empty vector pGEM3 and luciferase activities were measured 24 hours post-transfection.
(DOCX)

S1 Table Statistics for P-M2-1 docking and clustering with HADDOCK2.2 Webserver (S1 References 1,2). The top cluster is the most reliable according to HADDOCK.
(DOCX)

S1 References References for S1 Table. (DOCX)
