# Supplementary material for: RSV hijacks cellular protein phosphatase 1 to regulate M2-1 phosphorylation and viral transcription
Source: PLoS Pathog. 2018 Feb 28;14(3):e1006920. doi: 10.1371/journal.ppat.1006920 (PMC5847313; doi:10.1371/journal.ppat.1006920)
Supplement: S1 Fig — BSRT7/5 were transfected with pMT/Luc, pP or p-P-BFP, pL, pN and either pM2-1 or pM2-1-cherry, and Luc reporter activity was measured. (DOCX) [file ppat.1006920.s001.docx]

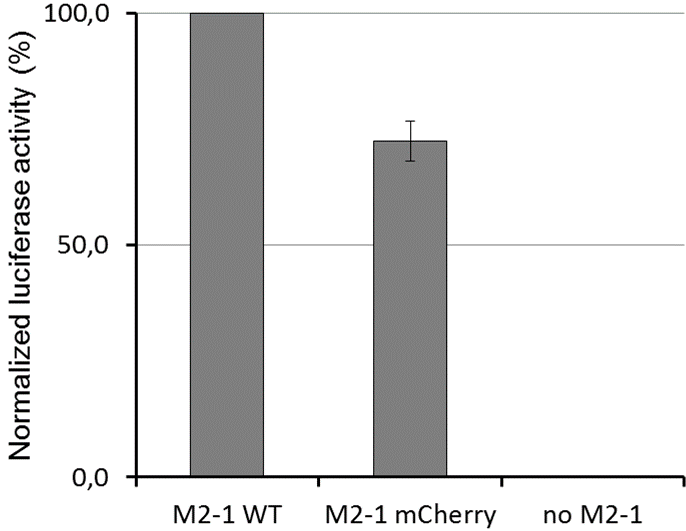


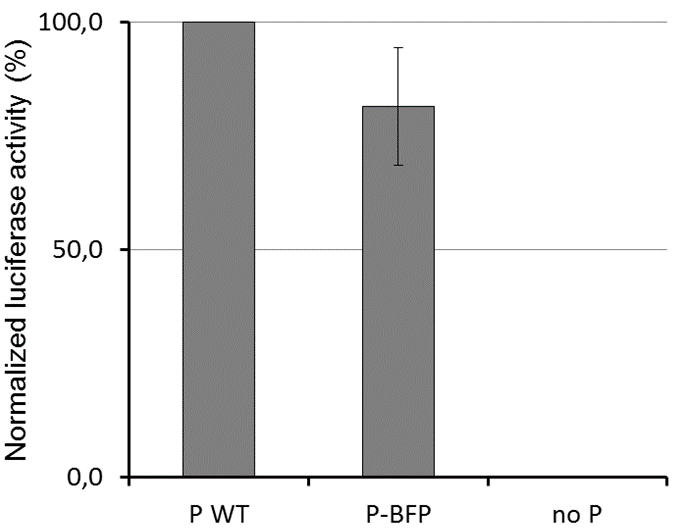


**S1 Fig. Effects of M2-1-mCherry and P-BFP for RSV polymerase activity.** BSRT7/5 were transfected with pM/Luc, pP or p-P-BFP, pL, pN and either pM2-1 or pM2-1-mCherry, and Luc reporter activity was measured.
