# Supplementary material for: RSV hijacks cellular protein phosphatase 1 to regulate M2-1 phosphorylation and viral transcription
Source: PLoS Pathog. 2018 Feb 28;14(3):e1006920. doi: 10.1371/journal.ppat.1006920 (PMC5847313; doi:10.1371/journal.ppat.1006920)
Supplement: S2 Fig — (Upper panel) Superimposed 1H-15N BEST-TROSY spectra of 25 μM 15N-labeled P[1–126] or 15N-labeled P, alone (magenta contours) and in the presence of 2 molar equivalents of GST-PP1α (black contours) are shown with residue-specific assignments. (Lower panel) Superimposed 1H-15N HSQC spectra of 10 μM 15N-labeled P[1–126], alone (green contours) and in the presence of 10 molar equivalents of GST (black contours). The buffer of the P[1–126]+GST sample contained 25 mM glutathione that yields natural abundance 15N signals indicated by stars. (DOCX) [file ppat.1006920.s002.docx]

**
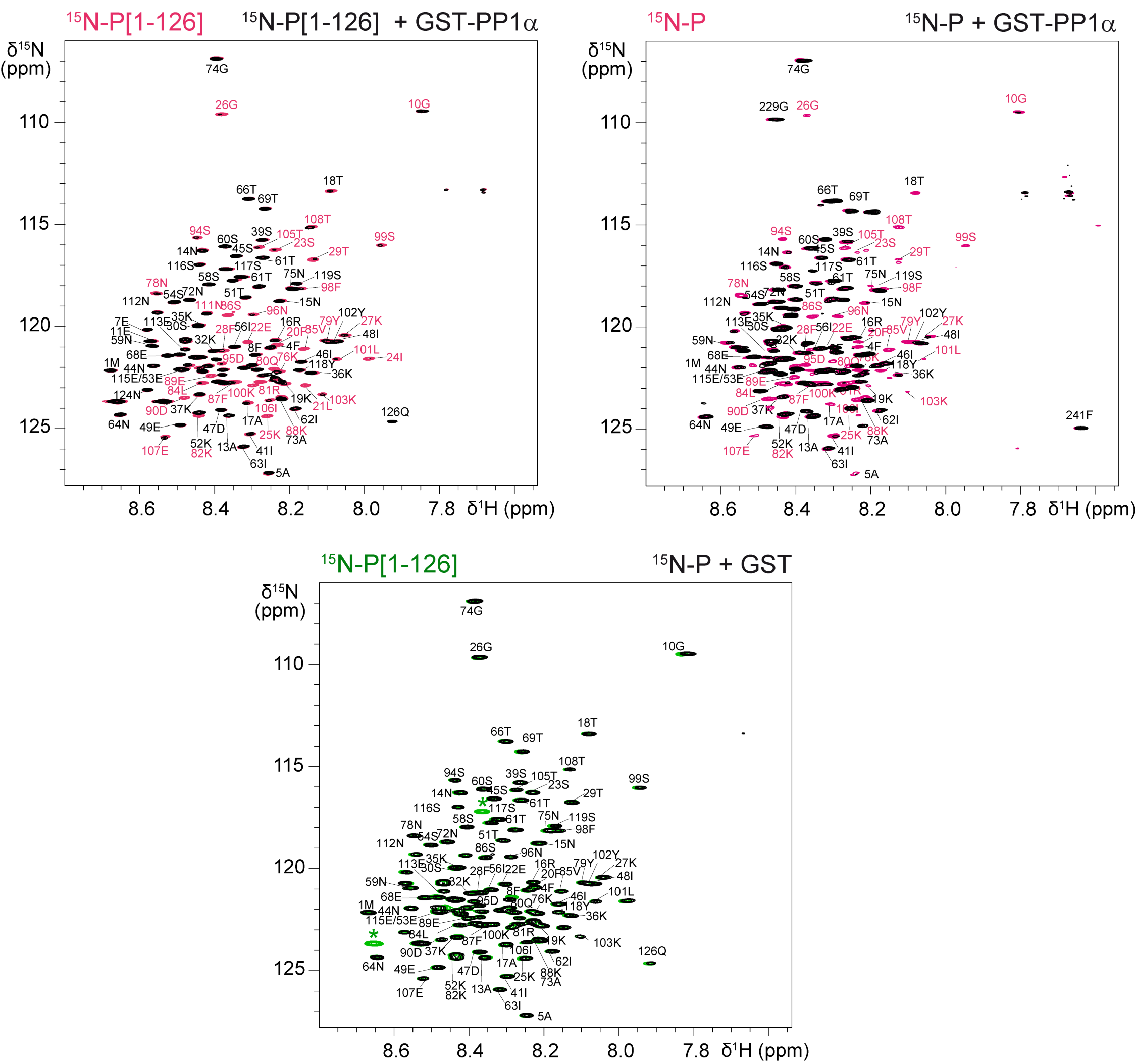
**

**S2 Fig. Perturbations of GST-PP1α in NMR spectra of P.** (Upper panel) Superimposed ^1^H-^15^N BEST-TROSY spectra of 25 µM ^15^N-labeled P[1-126] or ^15^N-labeled P, alone (magenta contours) and in the presence of 2 molar equivalents of GST-PP1α (black contours) are shown with residue-specific assignments. (Lower panel) Superimposed ^1^H-^15^N HSQC spectra of 10 µM ^15^N-labeled P[1-126], alone (green contours) and in the presence of 10 molar equivalents of GST (black contours). The buffer of the P[1-126]+GST sample contained 25 mM glutathione that yields natural abundance ^15^N signals indicated by stars.
