# Supplementary material for: RSV hijacks cellular protein phosphatase 1 to regulate M2-1 phosphorylation and viral transcription
Source: PLoS Pathog. 2018 Feb 28;14(3):e1006920. doi: 10.1371/journal.ppat.1006920 (PMC5847313; doi:10.1371/journal.ppat.1006920)
Supplement: S3 Fig — BSRT7/5 were transfected with pGaussia/Firefly minigenome vector, pP pL, pN and either pM2-1 or an empty vector pGEM3 and luciferase activities were measured 24 hours post-transfection. (DOCX) [file ppat.1006920.s003.docx]

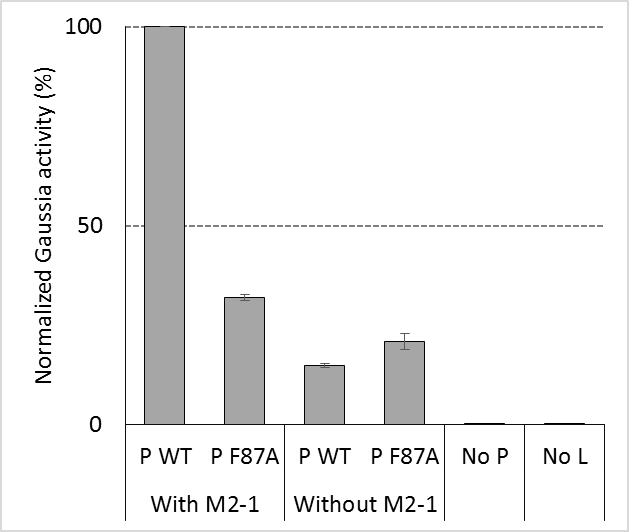


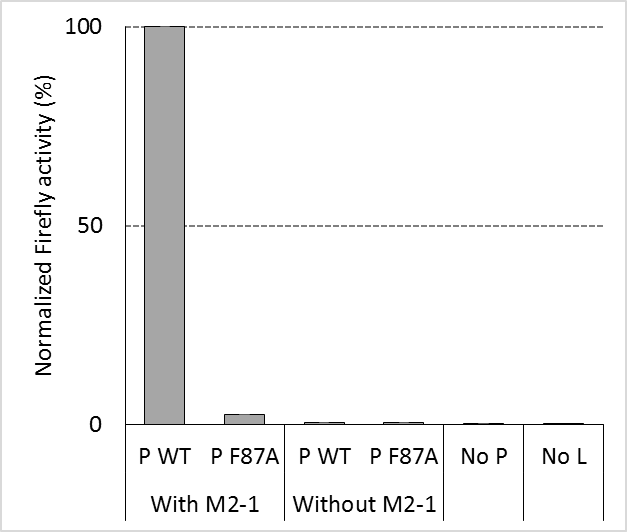


**S3 Fig. Comparative normalized activities of Gaussia and Firefly luciferase in the absence or presence of M2-1.** BSRT7/5 were transfected with pGaussia/Firefly minigenome vector, pP , pL, pN and either pM2-1 or an empty vector pGEM3 and luciferase activities were measured 24 hours post-transfection.
