# Supplementary material for: RSV hijacks cellular protein phosphatase 1 to regulate M2-1 phosphorylation and viral transcription
Source: PLoS Pathog. 2018 Feb 28;14(3):e1006920. doi: 10.1371/journal.ppat.1006920 (PMC5847313; doi:10.1371/journal.ppat.1006920)
Supplement: S1 Table — The top cluster is the most reliable according to HADDOCK. (DOCX) [file ppat.1006920.s004.docx]

| Cluster number | Haddock score  [a.u.] | Cluster size | RMSD from the overall lowest-energy structure  [Å] | Van der Waals energy  [kcal/mol] | Electrostatic energy [kcal/mol] | Desolvation energy [kcal/mol] | Restraints violation energy [kcal/mol] | Buried Surface Area  [Å^2^] | Z-Score |
| --- | --- | --- | --- | --- | --- | --- | --- | --- | --- |
| **1** | **-102.9 ± 2.1** | **101** | **0.7 ± 0.4** | **-40.4 ± 2.2** | **-155.5 ± 13.2** | **-33.6 ± 4.0** | **21.1 ± 13.91** | **1305.2 ± 28.1** | **-2,1** |
| 3 | -90.8 ± 4.8 | 19 | 3.0 ± 0.8 | -34.7 ± 2.9 | -149.5 ± 23.7 | -27.8 ± 3.9 | 15.7 ± 11.48 | 1216.8 ± 37.9 | -0.8 |
| 7 | -82.5 ± 16.0 | 4 | 5.7 ± 0.3 | -34.9 ± 4.8 | -138.7 ± 80.0 | -21.5 ± 2.4 | 16.9 ± 15.86 | 1159.0 ± 111.1 | 0.2 |
| 2 | -80.5 ± 5.3 | 24 | 7.3 ± 0.4 | -41.8 ± 2.9 | -25.1 ± 14.0 | -34.4 ± 4.0 | 7.1 ± 3.65 | 1267.9 ± 112.2 | 0.4 |
| 6 | -79.2 ± 5.1 | 9 | 5.8 ± 0.2 | -37.6 ± 8.0 | -146.9 ± 18.9 | -16.8 ± 1.3 | 45.8 ± 14.03 | 1316.3 ± 97.2 | 0.5 |
| 5 | -76.7 ± 8.7 | 10 | 6.8 ± 0.4 | -32.7 ± 8.1 | -140.8 ± 49.1 | -16.4 ± 7.4 | 6.3 ± 2.19 | 1258.6 ± 114.7 | 0.8 |
| 4 | -75.2 ± 4.1 | 18 | 7.7 ± 0.3 | -37.9 ± 6.0 | -59.9 ± 21.2 | -26.9 ± 8.3 | 15.7 ± 12.72 | 1228.6 ± 103.0 | 1.0 |
