# Supplementary material for: RSV hijacks cellular protein phosphatase 1 to regulate M2-1 phosphorylation and viral transcription
Source: PLoS Pathog. 2018 Feb 28;14(3):e1006920. doi: 10.1371/journal.ppat.1006920 (PMC5847313; doi:10.1371/journal.ppat.1006920)
Supplement: S1 References — (DOCX) [file ppat.1006920.s005.docx]

1 van Zundert, G. C. P. *et al.* The HADDOCK2.2 Web Server: User-Friendly Integrative Modeling of Biomolecular Complexes. *Journal of molecular biology* **428**, 720-725, doi:10.1016/j.jmb.2015.09.014 (2016).

2 Wassenaar, T. A. *et al.* WeNMR: Structural Biology on the Grid. *Journal of Grid Computing* **10**, 743-767 (2012).
